# miRNA-target network reveals miR-124as a key miRNA contributing to clear cell renal cell carcinoma aggressive behaviour by targeting CAV1 and FLOT1

**DOI:** 10.18632/oncotarget.3815

**Published:** 2015-04-14

**Authors:** Henriett Butz, Peter M. Szabó, Heba W.Z. Khella, Roy Nofech-Mozes, Attila Patocs, George M. Yousef

**Affiliations:** ^1^ Department of Laboratory Medicine and The Keenan Research Centre for Biomedical Science of St. Michael's Hospital, Toronto, Canada; ^2^ Department of Laboratory Medicine and Pathobiology, University of Toronto, Toronto, Canada; ^3^ Biometric Research Branch, Division of Cancer Treatment and Diagnosis, National Cancer Institute, National Institutes of Health, Bethesda, Maryland, USA; ^4^ HAS-SE “Lendulet” Hereditary Endocrine Tumors Research Group, Hungarian Academy of Sciences, Hungary

**Keywords:** renal cell carcinoma, miR-124-3p, integrated analysis, CAV1, FLOT1

## Abstract

Clear cell renal cell carcinoma (ccRCC) is an aggressive tumor with frequent metastatic rate and poor survival. Integrated analyses allow understanding the interplay between different levels of molecular alterations.

We integrated miRNA and gene expression data from 458 ccRCC and 254 normal kidney specimens to construct a miRNA-target interaction network.

We identified the downregulated miR-124-3p, -30a-5p and -200c-3p as the most influential miRNAs in RCC pathogenesis.miR-124-3p and miR-200c-3p expression showed association with patient survival, miR-30a-5p was downregulated in metastases compared to primary tumors. We used an independent set of 87 matched samples for validation. We confirmed the functional impact of these miRNAs by in vitro assays. Restoration of these miRNAs reduced migration, invasion and proliferation. miR-124-3p decreased the S phase of cell cycle, as well. We compared transcriptome profiling before and after miRNA overexpression, and validated CAV1 and FLOT1 as miR-124-3p targets. Patients with higher CAV1 and FLOT1 had lower miR-124-3p expression and shorter overall survival.

We hypothesize that these three miRNAs are fundamental contributing to ccRCC aggressive/metastatic behavior; and miR-124-3p especially has a key role through regulating CAV1 and FLOT1 expression. Restoration of the levels of these miRNAs could be considered as a potential therapeutic strategy for ccRCC.

## INTRODUCTION

Kidney cancer is a common urologic malignancy and its incidence has been steadily rising by 2–4% each year [1]. The vast majority of adult kidney cancers are renal cell carcinomas (RCC), and 70-85% of those are clear cell subtype (ccRCC) [2]. Around 30% patients are already at metastatic stage at the time of diagnosis even in the absence of symptoms [1]. Patients with metastatic disease have 13 months median survival and the 5 year survival rate under 10% [3]. MicroRNAs (miRNAs) are small, protein non-coding RNA molecules which post-transcriptionally regulate protein expression. Their role have been broadly investigated and demonstrated in several cancers including ccRCC [4].

Results of different studies for miRNA expression profiling in ccRCC show significant variations [5]. In order to overcome this obstacle, literature suggest that combining multiple datasets is more informative than considering them individually [6]. Is has also been suggested that increasing the number of replicates adds robustness to the experimental design and limits the undesired effects and bias in expression profiles, especially when comparing different platforms [7]. More recently, “integrated genomics” which compiles information from multiple levels of molecular changes, holds the promise of providing a better understanding of the interplay between molecular alterations [8].

In order to gain better understanding of the complex functional impact of miRNAs in ccRCC pathogenesis, we cross-matched miRNA and gene expression data from 458 ccRCC and 254 normal kidney specimens to construct a miRNA-target interaction network that can better reveal the involvement of miRNAs in kidney cancer. We further filtered our data by performing tissue-specific target prediction [9]. We identified the three most influential miRNAs in ccRCC and investigated their role by *in vitro* functional assays. We experimentally validated our results on independent sets of cases. We also confirmed the functional impact of these miRNAs by *in vitro* assays using cell line models. Comparing transcriptome profiling before and after miRNA overexpression, combined with pathway and gene ontology analysis, we identified critical targets and miRNA-mediated pathways with correlation to survival in ccRCC.

## RESULTS

### Construction of miRNA network of ccRCC

**Figure 1:**
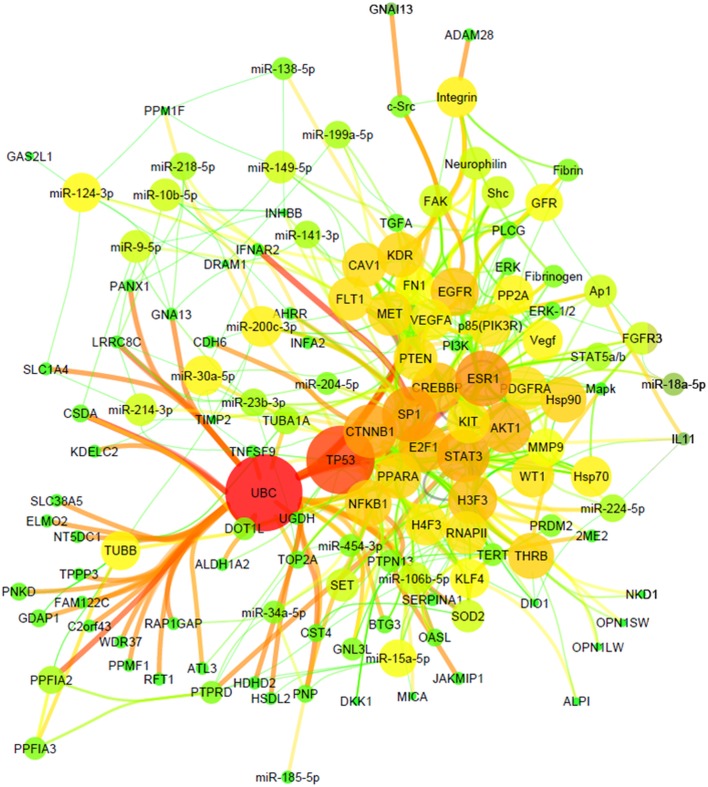
miRNA-target network of ccRCC Organic plot of miRNA-target network of ccRCC. Node's colour and size represents the number of interaction (node degree); edge's colour and size indicates the significance of interactions (edge betweeness).

Using the previously identified most common gene and miRNA alterations in ccRCC compared to normal kidney [10], we performed tissue-specific target prediction analysis. We identified 600 miRNAs-mRNA interaction pairs that consist of 49 miRNAs and 266 genes. In order to zoom in specifically to the involvement of miRNAs in ccRCC pathogenesis, we further filtered the target genes for the term “RCC associated genes” using Ingenuity Knowledge Database (Supplementary Table 1). Using these filtered interaction pairs we built multiple 35-node networks that are characteristic to RCC by functional annotation (Supplementary Table 2). We then merged the top three networks to obtain a 133-node miRNA network including 20 miRNAs and RCC-associated genes using Ingenuity Pathway Analysis Software. The network structure is formed of basic elements (genes and miRNAs; named nodes) and the connections representing miRNA-target interactions (named edges) (Figure 1). Our results show that miRNA effect on RCC has “divergent” properties where the same miRNA targets multiple genes. It is also “convergent” in nature, where multiple miRNAs have augmented effect on the same target. The most significant miRNAs (nodes with highest number of interactions) were miR-200c-3p, miR-124-3p and miR-30a-5p possessing the most connections, each having 7-9 targets (Table 1).

**Table 1:** miR-124-3p, miR-30a-5p andmiR-200c-3p targets in ccRCC miRNA network

miRNA Name	# of targets	Target gene name
miR-200c-3p	9	CDH6, CREBBP, FLT1, FN1, KDR, PPM1F, TIPM2, TUBB, VEGFA
miR-30a-5p	7	IFNAR2, KDELC2, LRRC8C, MET, TIMP2, TNFSF9, TP53
miR-124-3p	7	AHRR, CAV1, DRAM1, GAS2L1, GNA13, PPM1F, SLC1A4

### Experimental tissue validation and the potential significance of miR-124-3p, miR-200c-3p, and miRNA-30a-5p in ccRCC aggressiveness

**Figure 2:**
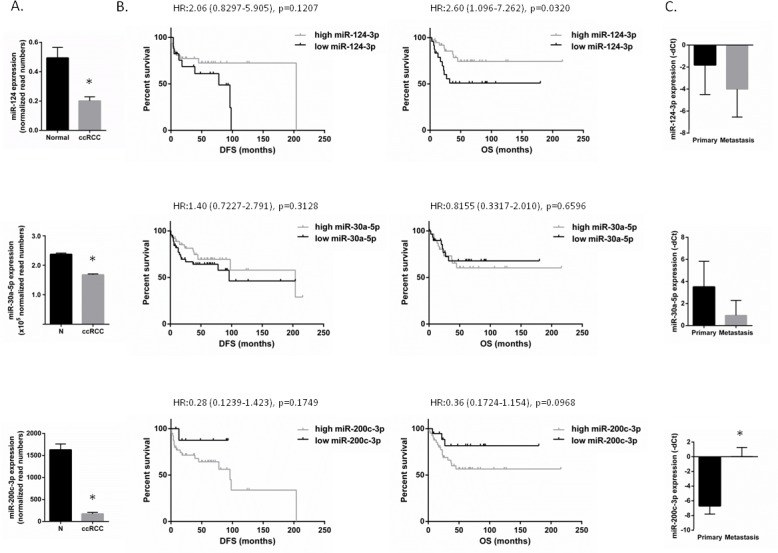
miR-124-3p, -30a-5p and -200c-3p expression in ccRCC and miRNA expression association with patient survival **A**: miR-124-3p, -30a-5p and -200c-3p are downregulated in ccRCC with -2.46, -1.41 and -9.39 fold change, respectively (*p* < 0.001) compared to normal kidney. N_Normal_: 66, N_ccRCC_: 499 – data were extracted from TCGA dataset. **B**: Survival analysis performed on our independent patient set of 62 ccRCCs. Patients having lower miR-124-3p or higher miR-200c-3p expression have worse disease-free and overall survival. There was no significant association between miR-30a-5p expression and survival. C: miRNA expression in primary ccRCC and matched metastatic specimens. miR-124 and -30a-5p were downregulated with 4.16 and 5.98 folds (*p* = 0.09 and *p* = 0.046). miR-200c-3p was overexpressed in metastases compared to the primary tumors (105 fold change; *p* < 0.0001). Columns and bars represent mean±SD, Stars (*) indicate statistical significance (*p* < 0.05).

All three miRNAs were downregulated in our analysis. We validated the dysregulation of these miRNAs and as it is shown in Figure 2, all three miRNAs were found to be downregulated in ccRCC compared to normal kidney.

Next, we examined the association of these miRNAs' expression and survival on an individual sample set of 62 cases of primary ccRCC and we compared the expression of these miRNAs in matched pairs of primary and metastatic ccRCC from the same patients

Higher expression of miR-124-3p was significantly associated with longer overall survival (HR:2.6; *p* = 0.032). This is in line with the tendency of lower expression of miR-124-3p (fold change: °4.61, *p* = 0.09) in metastatic tumors compared to primary ccRCC (Figure 2). Results did not reach statistical significance, probably due to the small sample size.

Interestingly, miR-200c-3p showed statistically significant higher expression in metastatic compared to primary tumors (fold change: 104.9, *p* < 0.001). miR-200c-3p showed an opposite pattern with higher expression levels associated with lower disease-free and overall survival, although this was not statistically significant.

There was no significant association between miRNA-30a-5p expression and survival (Figure 2). There was a tendency of lower expression of miR-30a-5p (fold change: °5.99, *p* = 0.07) in metastatic tumors compared to primary ccRCC.

We further validated our findings using an independent patient cohort from the TCGA database. Comparable results were obtained for all three miRNAs (Supplementary Figure 1).

### The global effect of miR-124-3p, miR-30a-5p and miR-200c-3p dysregulation

**Figure 3:**
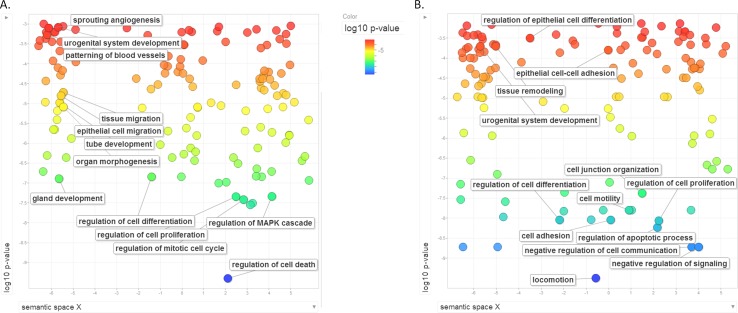
Gene Ontology (GO) analysis of ccRCC upon the effect of miR-124-3p, -30a-5p and -200c-3p **A**: Functional gene ontology clustering of ccRCC miRNA-target network members. **B**: Functional gene ontology analysis of miR-124, miR-30a-5p and miR-200c-3p targets evaluated by transcriptome profiling following triple-miRNAtransfection in 786-O and Caki-2 cells. Circles represent GO enriched categories. Colours and axis Y indicate log10(Benjamini-Hochberg FDR q-values).

We hypothesized that miRNAs (nodes) with the highest number of miRNA-target interactions (edges) will have the most significant effect on RCC pathogenesis. miR-200c-3p, miR-124-3p and miR-30a-5p were the most connected with 7-9 targets each. These were selected for further experimental validations. In order to investigate the functional impact of these miRNAs, we transfected all three miRNA mimics into 786-O and Caki-2 kidney cancer cell lines. Cell lines had been previously tested and showed relatively low expression of these miRNAs, and successful transfection was confirmed by RT-qPCR analysis (Supplementary Figure 2). We compared global gene expression before and after all three miRNAs co-transfection using microarray analysis. Significantly deregulated genes were subjected to IPA pathway analysis. The mostly altered pathways were “Molecular mechanism of cancer”, ”Integrin signaling”, ”Cell cycle”, and ”Regulation of the epithelial-mesenchymal transition pathway” (Supplementary Table 3). To gain more insight to the functional impact of these three miRNAs on ccRCC, we performed GO analysis of the genes significantly regulated as the result of triple transfection of these miRNAs. The most significantly altered functions were ”locomotion” and functional groups related to migration (such as ”cell adhesion”, ”cell motility”, ”cell-cell junction organization”, ”negative regulation of cell adhesion”. In order to examine if these three miRNAs are major players in ccRCC pathogenesis, we compared, using Gene Ontology (GO) analysis, our ccRCC miRNA network targets to the targets of these three miRNAs, as revealed by microarray analysis. As shown in Figure 3, there was a significant overlap in functional annotations of the two groups, indicating that these three miRNAs can be cardinal players in ccRCC pathogenesis.

These functions were further experimentally validated by PCR array analysis of the genes involved in extracellular matrix and cell adhesion pathways. Predicted targets of miR-124-3p were significantly downregulated upon its overexpression (Supplementary Figure 3). Overall, there was also a significant alteration in ECM and adhesion pathway signaling before and after miR-124-3p transfection (p <0.05) (Data not shown).

### Overall effect of miR-124-3p, miR-200c-3p, and miRNA-30a-5p on ccRCC cell migration, invasion and proliferation

**Figure 4:**
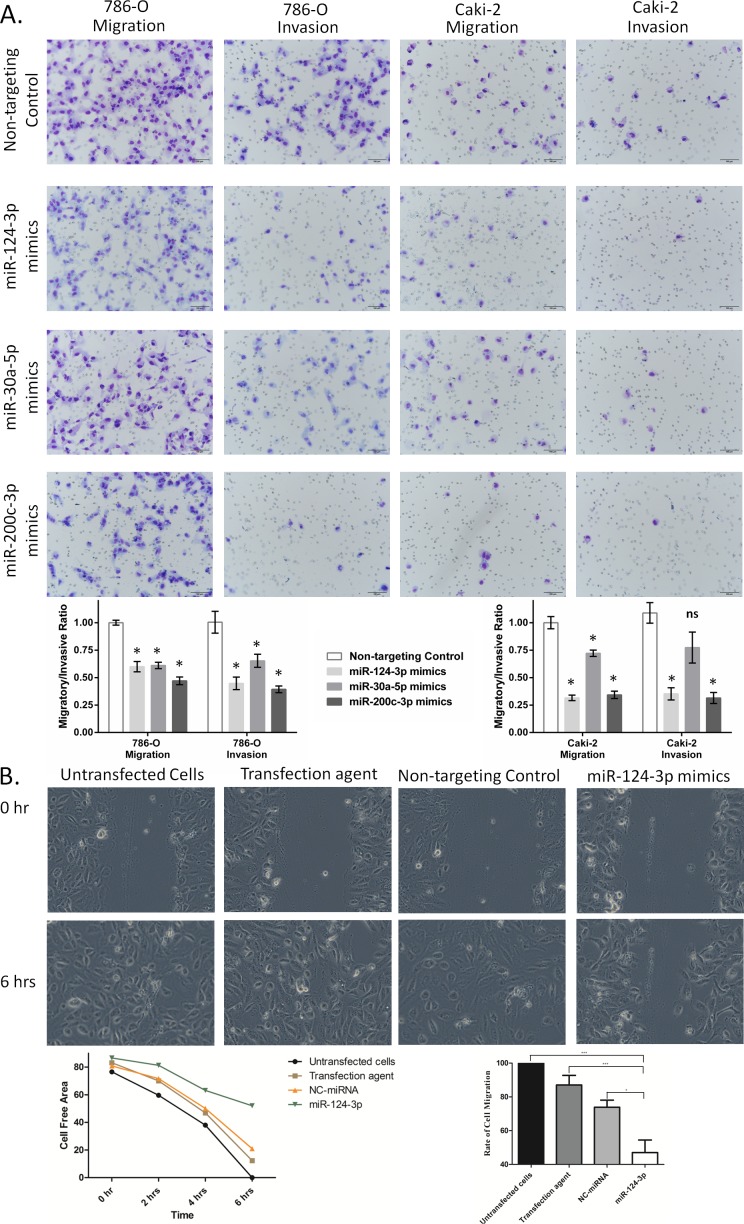
miR-124-3p, -30a-5p and -200c-3p effect on migration and invasion of kidney cancer cell lines **A**: miRNA effect on transwell migration and invasion of 786-O and Caki-2 cells. miR-124-3p, -30a-5p and 200c-3p overexpression decreased 786-O cells migration by 40%, 39% and 47%, respectively (*p* < 0.001), and reduced Caki-2 cell migration by 68%, 28% and 66%, respectively (*p* < 0.001). miR-124-3p and miR-200c-3p decreased cell invasion by 55% and 61%, respectively (*p* < 0.001) in 786-O cells and by 74% and 77% (*p* < 0.001) in Caki-2 cells. miR-30a-5p also inhibited invasion of 786-O cells by 35% (*p* < 0.001) but had no significant effect on Caki-2 cells (*p* = 0.07). Columns and bars represent mean±SEM. **B**: Representative photomicrographs showing the effect of miR-124-3p overexpression on the migration rate of the 786-O RCC cell line. The top row shows the cells at the time of wounding (0 h), and the bottom row shows cellular migration after 6 hours. Overexpression of miR-124-3p significantly decreased the rate of cell migration, with incomplete wound closure after 6h, compared to controls. Representative bar graph showing the effect of miR-124-3p on cellular migration.

GO analysis indicated that these miRNAs were involved in cell migration and invasion. We experimentally tested this by transwell migration and invasion assays 24 hours following miRNA mimics transfection. Restoration of levels of any of the three miRNAs decreased the transwell migration and invasion in 786-O cells (Figure 4). Overexpression of all three miRNAs significantly reduced migration and invasion of 786-O and Caki-2 cells, except miR-30a-5p which did result in significant reduction of Caki-2 cell invasion (Figure 4).

To further validate our results, we tested the effect of miR-124-3p on cell migration using wound healing assay. Hydroxyurea was used to inhibit proliferation. As shown in Figure 4, miR-124-3p transfection resulted in significant reduction of the rate of cell migration.

**Figure 5:**
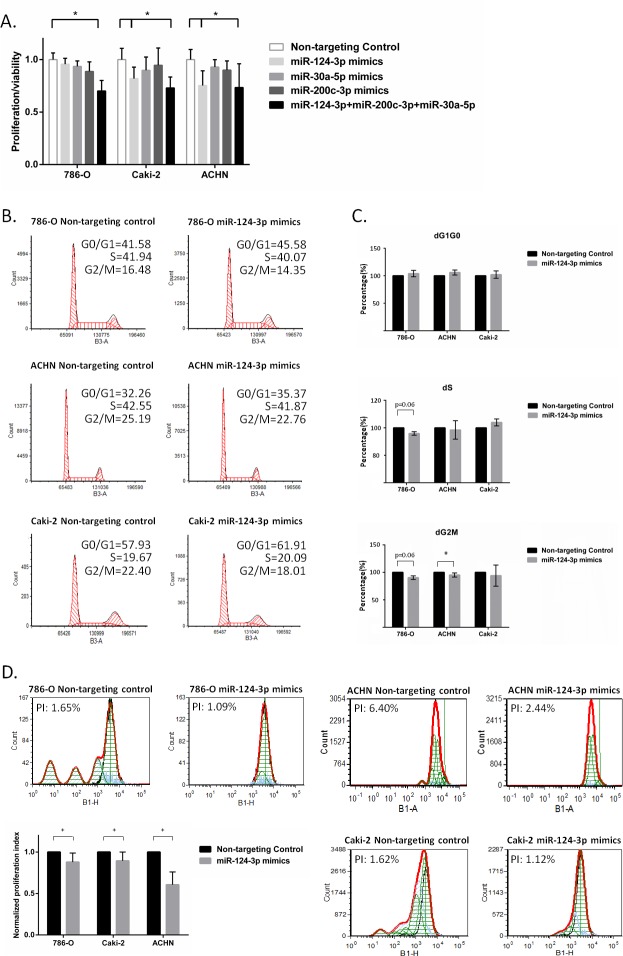
miR-124-3p, -30a-5p and -200c-3p effect proliferation and cell cycle **A**: Triple-miRNA transfection decreased cell proliferation/viability by 29.91%, 27.05% and 26.64% in 786-O, Caki-2 and ACHN cells using WST-1 assay. Individual miR-124-3p transfection also inhibited proliferation/viability of Caki-2 and ACHN cells by 18.12% and 24.75%. Columns and bars represent mean±SD, **B**: miR-124-3p mimic effect on cell cycle assessed by flow cytometry analysis. Representative pictures of cell cycle phase distribution after transfection with either Non-targeting Control or miR-124-3p mimic in different kidney cancer cell lines. **C**: normalized average dG1G0, dS and dG2M distribution (*N* = 3). **D**: miR-124-3p effect on proliferation investigated by CFSE flow cytometry analysis. Representative normalized CFSE dilution after transfection with averaged proliferation index, PI (*N* = 3). Data are presented as means of average percentage of cells ± SD. Stars (*) indicate statistical significance (*p* < 0.05), ns: non-significant.

We also assessed the effect of these miRNAs on cell proliferation/viability using WST-1 metabolic assay. Co-transfection of all three miRNAs at a 30 nM final concentration resulted insignificant decrease of cell proliferation/viability in 786-O, Caki-2 and ACHN kidney cancer cell lines. When individually transfected (30 nM), only miR-124 resulted in a small but significant decrease in cell proliferation in metastatic Caki-2 and ACHN cells. In the 786-O cells, there was a decrease in cell proliferation although this did not reach statistical significance (Figure 5A). Using MTT assay, miR-124-3p overexpression resulted in a slight decrease in the rate of cell proliferation. This did not, however, reach statistical significance (Supplementary Figure 4).

### Evaluation of individual miR-124 and miR-30a-5p function by gene expression profiling

Next, we wanted to zoom in to the specific functions of each of these three miRNAs. miR-200c-3p is a well-known tumor suppressor and as a member of miR-200 family controls epithelial-mesenchymal transition. Its role already has been documented in ccRCC [11, 12]. Therefore we selected miR-124-3p and miR-30a-5p for further investigation.

We first examined the effect of overexpression of each of these miRNAs on gene expression profile in the 786-O primary ccRCC cells using microarray analysis. miR-30a-5p overexpression resulted in significant alterations in a number of pathways including ”Cyclins and Cell Cycle Regulation” and ”Regulation of the Epithelial-Mesenchymal Transition Pathway” (Supplementary Table 4A). GO analysis showed the involvement of ”regulation of transcription” and ”TGF-β signaling” among others (Supplementary Table 4B).

For miR-124-3p, the most significant pathways were those related to migration and invasion, such as ”Regulation of Cellular Mechanics by Calpain Protease”, „Integrin signaling”, „Regulation of the Epithelial-Mesenchymal Transition Pathway”, „Actin Cytoskeleton Signaling”, „Epithelial Adherens Junction Signaling”, „Paxillin Signaling”, „Remodeling of Epithelial Adherens Junctions” (Supplementary Table 5A). Additionally, „Renal cell carcinoma signaling” and cell cycle-related pathways including ”G1/S Checkpoint Regulation” and ”Cyclins and Cell Cycle Regulation” were also significantly altered after miR-124-3p transfection. miR-124-3p targets in these pathways include MET (MET proto-oncogene, receptor tyrosine kinase), RRAS and RRAS2 (related RAS viral (r-ras) oncogene homolog and 2), GRB2 (growth factor receptor-bound protein 2), TGFB1 (transforming growth factor, beta 1) and RAP1A (RAP1A, member of RAS oncogene family). Similar results were obtained by GO analysis, where the most significant GO Biological Processes were „regulation of cellular component movement”, „nucleoside metabolic process” and „cell junction organization” (Supplementary Table 5B). As we already assessed cell migration and invasion after miR-124-3p mimics transfection as shown above, we investigated miR-124-3p influence on cell cycle and proliferation as a next step.

### miR-124-3p effect on cell cycle and proliferation

Despite we found the alteration of cell cycle and proliferation at transcriptional level assessed by microarray we could not clearly prove significant miR-124-3p effect on cell proliferation using two different metabolic assays. Therefore we performed flow cytometry, a more sensitive approach investigating miR-124-3p effect on cell cycle and proliferation.

We found an average 5% and 2% decrease in S phase and an average 10% and 5% decrease in G2M phase in 786-O and ACHN cells following 30 nM miR-124-3p transfection, however only S phase decrease was proved to be statistically significant in ACHN cells (Figure 5B-5C). Using CFSE flow cytometry proliferation assay, all three cell lines showed a small but significant decrease of proliferation index and present lower generation numbers after miR-124-3p transfection (Figure 5D).

**Figure 6:**
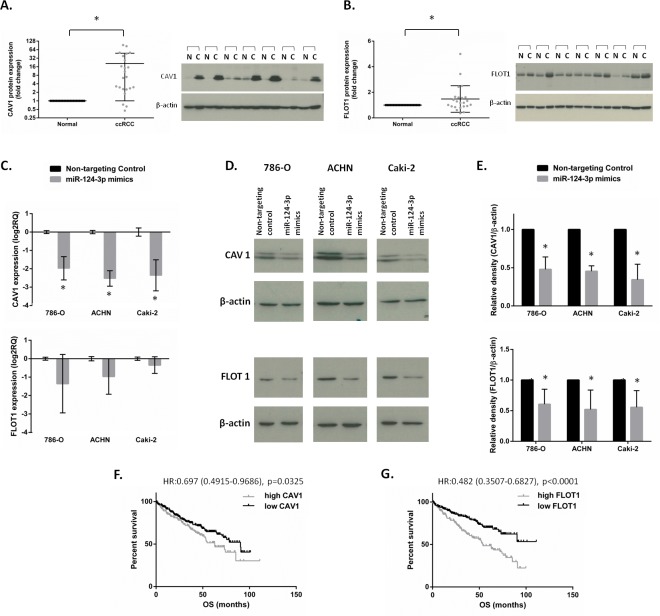
CAV1 and FLOT1 protein expression and association with survival in clinical specimens and validation as miR-124-3p targets miR-124-3p targets expression in ccRCC. Scatter plots show CAV1 (**A**) and FLOT1 (**B**) protein expression in 23 matched ccRCC vs. normal kidney specimens with representative western blot images. CAV1 and FLOT1 were upregulated with 21 and 1.5 folds, respectively (*p* < 0.01 and *p* = 0.03) in tumour tissues compared to their matched normals. **C**: miR-124-3p transfection resulted in CAV1 mRNA downregulation with 3.9, 5.8 and 5.11 folds (*p* < 0.001) in 786-O, ACHN and Caki-2 cells, respectively. FLOT1 expression was also reduced but this did not reach statistical significance. **D**-**E**: Both CAV1 and FLOT1 protein were downregulated following miRNA transfection by 52%, 55%, 66% (*p* < 0.001) and 39%, 48%, 44% (*p* < 0.05) in 786-O, ACHN and Caki-2 cells, respectively. F-G: Kaplan-Meier analysis showing that higher CAV1 or FLOT1 expressions are associated with worse survival of ccRCC patients. Columns and bars represent mean±SD, stars (*) indicate statistical significance (*p* < 0.05).

### Target validation

Because miR-124-3p accomplished only a slight effect on cell proliferation next we focused its influence on migration/invasion processes. To understand the mechanism by which miR-124-3p can affect tumor cell migration and invasion, we selected two targets of miR-124-3p involved in cell migration-invasion for further validation. Caveolin 1 (CAV1) was downregulated by microarray with −4.59 and −8.00 fold change (*p* < 0.001 and =0.007) following miR-124-3p transfection, and it was involved in pathways related to migration (Supplementary Table 5A). Flotillin 1 (FLOT1) is documented in the literature to be implicated in cell migration and was a validated target of miR-124-3p in breast cancer [13]. We investigated CAV1 and FLOT1 protein expression by Western blot in 23 pairs of normal-ccRCC specimens, and found CAV1 protein to be upregulated with an average 20.8 fold and FLOT1 with 1.47 fold in ccRCC compared to normal counterparts form the same patient (Figure 6A-6B). As miRNAs having a so-called fine tuning effect on the target expression 1,47 fold change on protein level can be resulted as a miRNA effect. In many cases miRNAs do not lead to expression change on mRNA level but only on protein level. To include these possibilities as well we selected FLOT1 as a potential second target beside CAV1. We further assessed the effect of miR-124-3p overexpression on these two targets at the mRNA level (by RT-qPCR) and protein level (by Western blot analysis) in 786-O, ACHN and Caki-2 cell lines. miR-124-3p overexpression resulted in a significant reduction of the expression of CAV1 at the mRNA level (Figure 6C) and the expression of CAV1 and FLOT1 at protein level (Figure 6D-6E).

We also investigated the association between patient survival and CAV1, FLOT1 expression. Compared to miR-124-3p, opposite pattern was seen with CAV1 and FLOT1: higher expression associated with worse overall survival of ccRCC patients (HR:0.697; *p* = 0.0325 and HR:0.483; *p* < 0.001) (Figure 6F-6G).

To further examine if the effect of miR-124-3p can be mediated through CAV1 and FLOT1, we tested the effect of knocking down of these two molecules (by siRNAs) on cell migration. CAV1 and FLOT1 siRNA knock down resulted in a similar effect on cell migration, suggesting that miR-124 effect can be mediated through these targets (Supplemetary Figure 5).

## DISCUSSION

To identify the most significant miRNAs and to understand their contribution to ccRCC, we constructed a miRNA-gene interaction network. Because target prediction is not always accurate, we added a number of unique features to improve accuracy of our network, including using tissue-specific target prediction, and filtering the targets for “RCC associated genes”. We identified miR-124-3p, -30a-5p and -200c-3p as the most influential miRNAs by having the highest number of interactions. The downregulation of these miRNAs is in keeping with previous results from our lab and others [5]. We also recognized their roles in aggressive/metastatic behaviour by *in vitro* migration and invasion assays and gene expression profiling.

Based on our findings miR-124-3p is a tumor suppressor miRNAs as it is downregulated in ccRCC compared to normal kidney; and patients having lower miR-124-3p expression demonstrate worse disease-free and overall survival. Also miR-124-3p shows a tendency of lower expression in metastatic specimens compared to primary tumors. This is further confirmed by our experimental evidence showing its significant suppressor effect on migration, invasion and a slight effect on proliferation of different type of kidney cancer cell lines.

Interestingly miR-200c-3p showed an opposite correlation to patient survival, where patients having higher expression had worse DFS and OS. This can be related to the fact that in metastatic samples miR-200c-3p showed higher expression compared to primary tumors. Other studies, however, showed that miR-200c-3p decreases the metastatic ability of renal carcinoma cells by upregulating E-cadherin through ZEB1 [14, 15, 5]. Thus the involvement of miR-200c-3p in tumor progression needs to be investigated further. miR-30a-5p inhibited migration and invasion of 786-O primary cells and migration of the metastatic Caki-2 cells, and this correlated with its lower expression in metastatic compared to primary tumors. Overall, our results show that these three miRNAs together can possibly have a key role in ccRCC.

To our knowledge, this is the first report validating CAV1 and FLOT1 as miR-124-3p targets in ccRCC. We also showed that these proteins are overexpressed in ccRCC and patients having higher CAV1 and FLOT1 expression present worse overall survival, with an inverse correlation to the survival outcome of miR-124-3p which emphasizes the role of the miRNA in controlling them.

Our results showing miR-124 downregulation in cancer is not unprecedented. miR-124 was reported as tumor suppressor miRNA in several cancers including prostate, bladder, breast, colorectal, nasopharyngeal, gastric, pancreatic or hepatocellular cancers. It was documented to target Collagen prolyl-hydroxylases [16], ROCK1 (Rho-associated, coiled-coil containing protein kinase 1) [17], ETS1 (v-ets avian erythroblastosis virus E26 oncogene homolog 1) [18], KITENIN (KAI1 C-Terminal Interacting Tetraspanin) [19], FOXQ1 (forkhead box Q1) [20], EZH2 (enhancer of zeste 2 polycomb repressive complex 2 subunit) [21], RAC1 (rho family, small GTP binding protein Rac1) [22] or STAT3 (signal transducer and activator of transcription 3) [23]. Majority if not all of these molecules are implicated in cell migration-invasion and proliferation. In glioma and pancreatic cancer, the connection between miR-124 downregulation and worse patient survival were also reported [22, 24].

CAV1 and FLOT1 were also shown to affect migration and invasion in porcine kidney epithelial cells and breast cancer [13, 25]. CAV1 is a main component of the plasma membrane caveolae and influence caveolae-mediated endocytosis [26]. Caveolin-1 can induce lamellipodia formation via an Akt-dependent pathway [27], implying its role in cell migration and invasion. It also functions as a membrane adaptor to link the integrin alpha subunit to the tyrosine kinase Fyn. Upon integrin ligation, Fyn is activated and promotes cell cycle progression through Ras-ERK pathway [28]. CAV1 also inhibits anoikis, a form of programmed cell death which is induced by anchorage-dependent cells detaching [29]. It is frequently overexpressed in different neoplasms such as prostate [30], bladder [31], and pancreatic cancers [32]. In ccRCC, it was reported to be overexpressed [33, 34], and to be associated with poor prognosis [35].

Flotillin-1 (FLOT1), similarly to CAV1 is also related to endocytosis and vesicular trafficking [36]. It was found to upregulated in several types of cancer as breast [37], esophageal [38], hepatocellular [39] and non-small cell lung cancer [40]. FLOT1 upregulation was associated with tumor progression and patient survival. Recently it was found that FLOT1 has a direct role in epidermal growth factor (tyrosine kinase) receptor activation and the latter MAP kinases activation [41] which can explain its oncogenic role in tumorous tissues. During the manuscript preparation, a report was published showing that higher expression of FLOT1 is associated with cancer progression and poor patient survival in ccRCC [42] which is consistent with our results. It should be also noted that there are other important targets and pathways that merit investigation in future studies.

In conclusion, we provide evidence showing the presence of a miRNA-target network that significantly contributes to ccRCC pathogenesis. miR-124-3p, -30a-5p and -200c-3p are key miRNAs participating in ccRCC aggressive/metastatic behaviour; and miR-124-3p has a key role in this - at least in part - through regulating CAV1 and FLOT1 expression. Restoration of the levels of these tumor suppressor miRNAs could be considered as a potential therapeutic strategy for RCC.

## MATERIALS AND METHODS

### Datasets and network construction

Using our previously assembled data (1531 and 49 most significantly dysregulated genes and miRNAs in ccRCC [10]) based on 593 ccRCC and 389 normal kidney specimens, we performed tissue-specific target prediction as previously described [9]. In order to ascertain the role of miRNAs in ccRCC pathogenesis we filtered the target genes for the term “RCC associated genes” using Ingenuity Knowledge Database, which is a curated database having over 10.000 peer-reviewed citations (http://www.ingenuity.com/products/ipa) and contains genes have previously been described as altered related to RCC. We visualized our network using Cytoscape 3.1.0. software, mapping node's colour and size to degree and edge's color and size to edge betweeness. Betweeness centrality of an edge is the sum of the fraction of all-pairs shortest paths that pass through it. An edge with high edge betweeness centrality score represents a bridge-like, or bottleneck connector between different parts of the network.

### Validation set patient cohorts

The study was approved by the Research Ethics Board of St. Michael's Hospital. Retrospectively formalin-fixed, paraffin-embedded (FFPE) tumor samples of 62 ccRCC patients were obtained from St. Michael's Hospital and analyzed for miRNA expression. 6 primary and metastatic matched FFPE specimen pairs were investigated. Additionally fresh tissue of 23-23 paired normal kidney and ccRCC specimens were also collected for protein analysis. Routine histopathology diagnoses were confirmed by 2 independent pathologists. Descriptive statistics of the patient cohort is described in Supplementary Table 6.

### Validation on TCGA database

We also validated our results using the publicly available ccRCC dataset of The Cancer Genome Atlas (TCGA), retrieving the survival data through the cBio Cancer Genomics Portal. In total, we downloaded and analysed 499 ccRCC and 66 normal samples miRNA sequencing data. To compare the miRNA expression *T*-test with Welch correction was used by GraphPad Prism software. In total, data of 425 patients were available for analysis. Optimal cut off values were identified using method “outcome euclidean” algorithm by CutoffFinder [43], survival data was then analysed by the Kaplan-Meier method, followed by log-rank test.

### miRNA mimics transfection

Primary 786-O and metastatic ACHN and Caki-2 kidney cancer cell lines were purchased from ATCC. Cells were transfected with 30 nM any of miR-124-3p, miR-30a-5p, miR-200c-3p mirVana™ miRNA Mimics (ID: MC10691, MC11062, MC11714) or with Non-targeting Control miRNA mimic #1 (#:44640633, Life Technologies, Grand Island, NY, USA) using LipofectamineRNAiMAX (#13778075, Life Technologies) as previously described [10]. Transfections were controlled by RT-qPCR from extracted RNA after 24h using TaqMan assays (Supplementary Figure 2).

### RNA extraction from cell lysate and FFPE samples

Total RNA extraction from cells for RT-qPCR and microarray analysis was performed using (miRNeasy kit, #217004, Qiagen, Mississauga, Canada). For FFPE samples RNA isolation was done using six 1 mm cores of pure tumor areas of ccRCC and metastases, as we previously described [44] using the miRNeasy FFPE Kit (217504, Qiagen, Mississauga, Canada). RNA concentrations and quality were determined using NanoDrop (Thermo Scientific, Hudson, NH, USA) and Agilent 2100 Bioanalyzer (Agilent, Santa Clara, CA, USA).

### Gene expression profiling by microarray

Two hundred nanograms of total RNA were labelled using Illumina TotalPrep-96 RNA Amplification kit (PN:4393543, Ambion Life Technologies, Grand Island, NY, USA) as per amplification protocol. 750ng of cRNA were generated and hybridized into one Human HT-12 V4 BeadChip. The BeadChip was incubated at 58°C, with rotation speed 5 for 18 hrs for hybridization. The BeadChip was washed and stained as per Illumina protocol and scanned on the iScan (Illumina, San Diego, CA, USA). All samples passed Illumina sample dependent and independent QC Metrics. Data analysis was performed by Genespring GX 12 Software (Agilent Tech Inc, Santa Clara, CA, USA). Raw data was filtered by percentile (lower cut-off: 20). Fold change filter was set to 2-fold, and then unpaired *t*-test was used to identify significant (*p* < 0.05) gene expression changes with multiple testing correction (Benjamini-Hochberg) to control the false discovery rate and get statistically reliable results. Functional analysis were done by IPA pathway analysis and GO categories of miRNA target genes were determined using Generic Gene Ontology (GO) Term Mapper, which were then submitted to REVIGO [45] for further analysis.

### TaqMan^®^ Array human gene expression assays

We tested the effect of miR-124-3p overexpression on extracellular matrix and cell adhesion pathway by TaqMan^®^ Array human gene expression assaysusing the Step One™ Plus Real-Time PCR System (Applied Biosystems). ACHN cells were transfected with miR-124-3p and compared to cells transfected with a non-targeting control miRNA. Total RNA was isolated using the RNeasy Mini Kit (Qiagen) according to the manufacture's protocol. Reverse transcription was done by using High-Capacity cDNA Reverse Transcription Kit (Applied Biosystems, Foster City, CA). This was followed by performing PCR amplification using the TaqMan^®^ Array human gene expression assays specific for human extracellular matrix and cell adhesion molecules. Fold change was calculated by 2^(−ΔΔCt)^. First ΔCt were calculated for each target by normalizing its threshold cycle to the average of endogenous controls including 18S, *GAPDH, HPRT1, GUSB*,*RPLP0 and PPIA*. Then ΔΔCt was calculated as follows ΔΔCt = [ACHNcells transfected with miR-124-3pΔ;Ct - ACHN cells transfected with a non-targeting control miRNAΔCt].

### RT-qPCR of miRNAs and miRNA target genes

For miRNA analyses, reverse transcription was performed with 1 ng total RNA using the TaqMan MicroRNA Reverse Transcription Kit; miRNA expression was measured on Viia 7 Real-Time PCR System (Life Technologies, Grand Island, NY, USA) with TaqMan microRNA Assays for miR-124-3p, miR-30a-5p and miR-200c-3p (Assay ID: 001182, 000417, 002300) as previously described [46]. Relative expression was determined using the ddCt method and expression values were normalized to the geometric mean of RNU44 and 48 (Assay ID: 001094, 001006) [44, 47]. miRNA expression comparison was carried out by paired T test, survival data was analyzed by the Kaplan-Meier method after determining cut-offs by CutoffFinder and followed by log-rank test.

For evaluation of target genes expression 1 ug total RNA was reversed transcribed using the High Capacity cDNA Reverse Transcription Kit (Life Technologies, Grand Island, NY, USA). Gene expression was measured using Fast Syber Green Master Mix (Life Technologies, Grand Island, NY, USA) using the following primers: CAV1 F-5′CCGCGACCCTAAACACCTC-3′, R-5′GCCTTCCAAATGCCGTCAA-3′; FLOT1 F-5′ACATTGCCCTGGAGACGTTAG-3′, R-5′ACACTGATGCCCATGTTGAC-3′. The combination of peptidylprolylisomerase A (PPIA) and Tubulin-α (TUBA1A) was used as an endogenous control: PPIA, F–5′-ATGCTGGCCCCAACACAA-3′, R–5′-CCCTCTTTCACCTTGCCACC-3′; TUBA1A, F–5′-TCTTCCACCCTGAGCAACTT-3′, R–5′-CTCCAGCTTGGACTTCTTGC-3′ as previously described [10].

### Viability, migration and invasion assays

Following miRNA mimics transfection, viability, migration and invasion assays were conducted as previously described [48]. Briefly, 48h after 30 nM miRNA mimics transfection cell viability and proliferation was monitored by WST-1 Cell Proliferation Reagent (#05015944001, Roche Applied Science, Indianapolis, IN, USA) following the manufacturer's protocol. Cell behaviour was investigated by transwell migration and invasion assays (#354578 and #354480, BD-Biocoat, Bedford, MA, USA) on 8.0 μm chambers 24 hours following transfection as previously published [44]. Hydroxyurea was added to inhibit cell proliferation during migration and invasion assays. Statistical significance was calculated using one-way ANOVA with Bonferroni's multiple comparisons test.

Migration was additionally assessed by wound healing assay. Cells were seeded in a 12 well plate, and transfected either with Lipofectamine RNAiMAX Transfection Reagent, miR-124-3p, non-targeting control miRNA, siRNAs specific for CAV1 (siRNA ID: s2448 Cat#: 4390824, Life technologies), or FLOT1 (siRNA ID: s19915, Cat#: 4392420), or non-targeting control siRNA (Cat#: 4390843). Twenty four hours after transfection, the cell monolayer was wounded using a 200μL pipette tip. Photomicrographs were taken every 30 minutes starting at the time of wounding (0 hrs) and continued up to 8 hrs using a microscope in an incubation chamber with 37ºC and 5% CO_2_. The microscope was programmed to take a series of photomicrographs at the exact place. Image J Software (National Institutes of Health, Bethesda, ME, http://rsbweb.nih.gov/ij/) was used for cell migration analysis. Percent cell-free area was calculated as [(cell-free area_8hrs_/cell-free area_0hrs_) x 100] and cell migration rate was displayed as the percent of cell covered area (100 - percent cell-free area).

### Protein extraction and western blotting

From fresh tissue samples total protein was extracted as previously described [49]. Briefly, tissues were homogenized using a hand-held homogenizer in ice-cold phosphate buffered saline (PBS) with 1:100 protease-inhibitor cocktail (#P3840, Sigma-Aldrich, St Louis, MO, USA). Cell debris was then removed by centrifugation (for 30 min at 4°C, 14,000 rpm), then the supernatant was used for analysis.

48h after transfection cells were lysed on ice using cold lysis buffer (100 mMNaCl, 30 mM Hepes (pH 7.5), 20 mMNaF, 1 mM EGTA, 1% Triton X-100, supplemented with 1 mM Na3VO4, 1 mM PMSF, and 1:100 protease-inhibitor cocktail). Protein concentration was determined by BCA protein assay (Pierce Biotechnology, Rockford, IL, USA). Total protein was separated in 10% SDS–PAGE, transferred to a nitrocellulose membrane and incubated with 1:1000 primary rabbit antibodies (CAV1, #3238; Flotillin-1, #3253; Cell Signalling Technology Inc., Danvers, MA, USA) overnight. Membranes were stripped and re-probed for mouse anti-β-actin (1:1000, Cell Signalling Technology Inc.) as a loading control. Anti-mouse and anti-rabbit-HRP conjugated IgGs were used as secondary antibodies (1:10000, W402B, W401B, Promega, Madison, WI, USA). Band intensities were quantified using Image J software (Bethesda, MD, USA). For comparison of normal and ccRCC samples and for calculating p-value the Wilcoxon Signed Ranks Test was applied.

### Cell cycle analysis

48h following transfection 786-O, Caki-2 and ACHN cells were harvested, washed with 1xPBS and then fixed with ice cold 70% ethanol for 15 min on ice. Cells were then washed with PBS and cell pellet was resuspended in 20 mg/ml RNAse A solution (#:LS005649, Worthington Biochem, Lakewood, NJ, USA) and incubated for 1 hour at 37ºC. Propidium Iodide (#:556463, BD BioSciences) was added at a final concentration 50 ug/ml and incubated overnight at 4^o^C. DNA content was analysed by flow cytometry on a MACS Quant flow cytometer (MiltenyiBiotec, Auburn CA) at an event rate of < 500 evs/s. Data was analysed using Multicycle AV (FCS Express, DeNovo Software, Los Angeles, CA, USA) and evaluated for acceptable G1 peak CV and model fit chi squared values with appropriate singlet gating to exclude coincident events. *P*-value was calculated using the Wilcoxon Signed Ranks Test.

### CFSE proliferation analysis

The effect of miR-124-3p on cell proliferation was analysed by carboxy fluorescein diacetate succinimidyl ester (CFSE, #422701, BioLegend Inc, San Diego, CA, USA) dye dilution using flow cytometry as previously described [50], with minor modification. Briefly, 786-O, Caki-2 and ACHN cells were labelled with CFSE dye according to the manufacturer instructions. Cells were trypsinized and suspended in in 1xPBS at a 5×10^6^ cells/ml. Cells were mixed with CFSE at a final concentration 1 uM for 10 min. Reactions were quenched with 10 ml ice-cold complete media and washed twice. Cells then were seeded at a density of 4.5×10^6^ cells/60 cm^2^ dish, and transfected 24h later with 30 nM miR-124-3p mimics or Non-targeting Control as described above. After 48 h cells were trypsinized, washed with PBS and CFSE dilution was determined by flow cytometry using a MACS Quant (MiltenyiBiotec, Auburn, CA, USA). The acquired data were analysed by FCS Express 4 (DeNovo Software, Los Angeles, CA, USA) based on live, single cell gates followed by proliferation analysis to model the generation peaks and proliferation index of each population. P value was calculated using the Wilcoxon Signed Ranks Test.

## SUPPLEMENTARY MATERIALS FIGURES AND TABLES


