# Supplementary material for: miRNA-target network reveals miR-124as a key miRNA contributing to clear cell renal cell carcinoma aggressive behaviour by targeting CAV1 and FLOT1
Source: Oncotarget. 2015 Apr 14;6(14):12543–57. doi: 10.18632/oncotarget.3815 (PMC4494957; doi:10.18632/oncotarget.3815)
Supplement: Supplementary file 1 [file oncotarget-06-12543-s001.pdf]

miRNA-target network reveals miR-124as a key miRNA contributing to clear cell renal cell carcinoma aggressive behaviour by targeting CAV1 and FLOT1

Supplementary Material

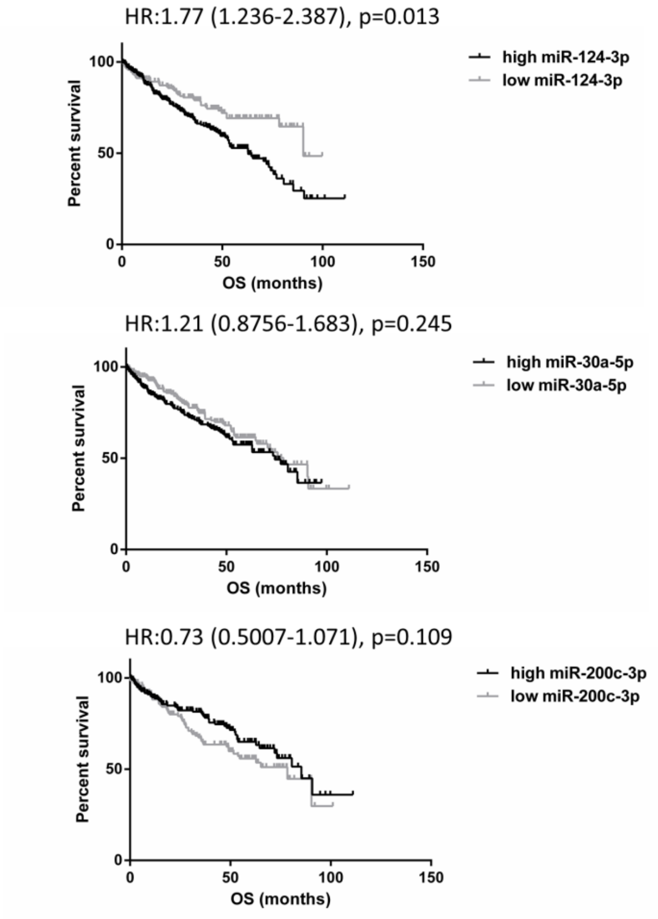

Supplementary Figure 1: miR-124-3p, -30a-5p and -200c-3p association with patient survival on TCGA dataset

Lower miR-124-3p expression associated with worse overall survival in patients having ccRCC (data extracted from TCGA database). There was no significant association between miR-30a-5p or miR-200c expression and overall survival.

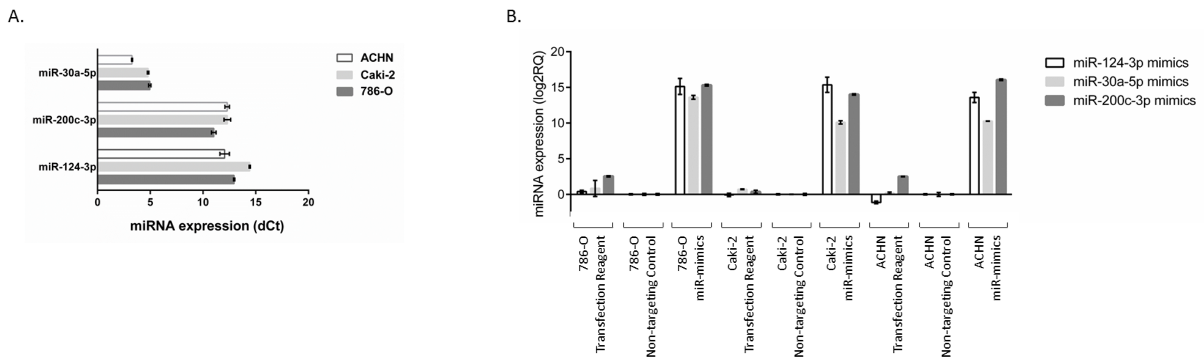

Supplementary Figure 2. miR-124-3p, -30a-5p and -200c-3p endogenous expression in kidney cancer cell lines; and miRNA mimics transfection evaluation

A: miRNA expression was quantified in ACHN, Caki-2 and 786-O cell lines. **B:** miRNA mimics transfections were evaluated by RT-qPCR using TaqMan assays after 24hposttransfection.

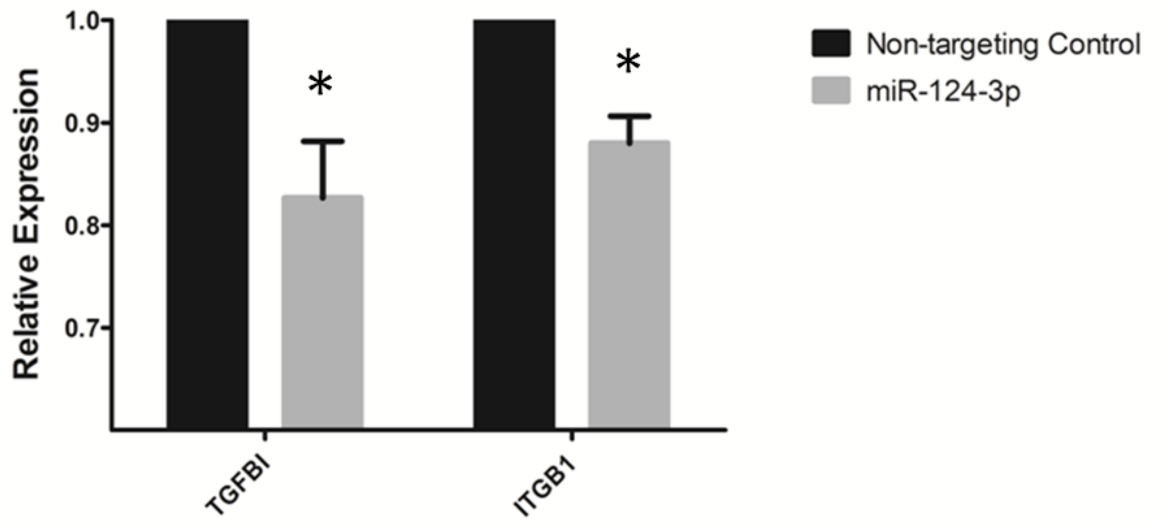

Supplementary Figure 3. Validation of miR-124-3p targets

A representative bar graph showing miR-124-3p decreased the expression levels of key molecules involved in extracellular matrix and cell adhesion pathways including *TGFBI* and *ITGB1*.

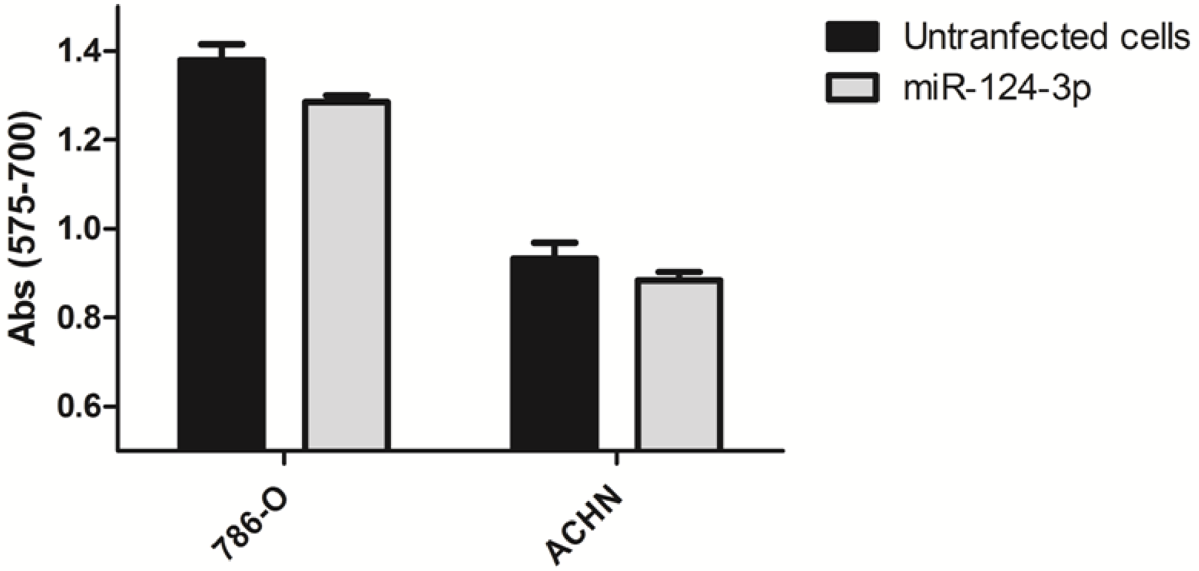

Supplementary Figure 4. miR-124-3p effect on cell proliferation

miR-124-3p overexpression resulted in a slight decrease in the rate of cell proliferation. This did not, however, reach statistical significance.

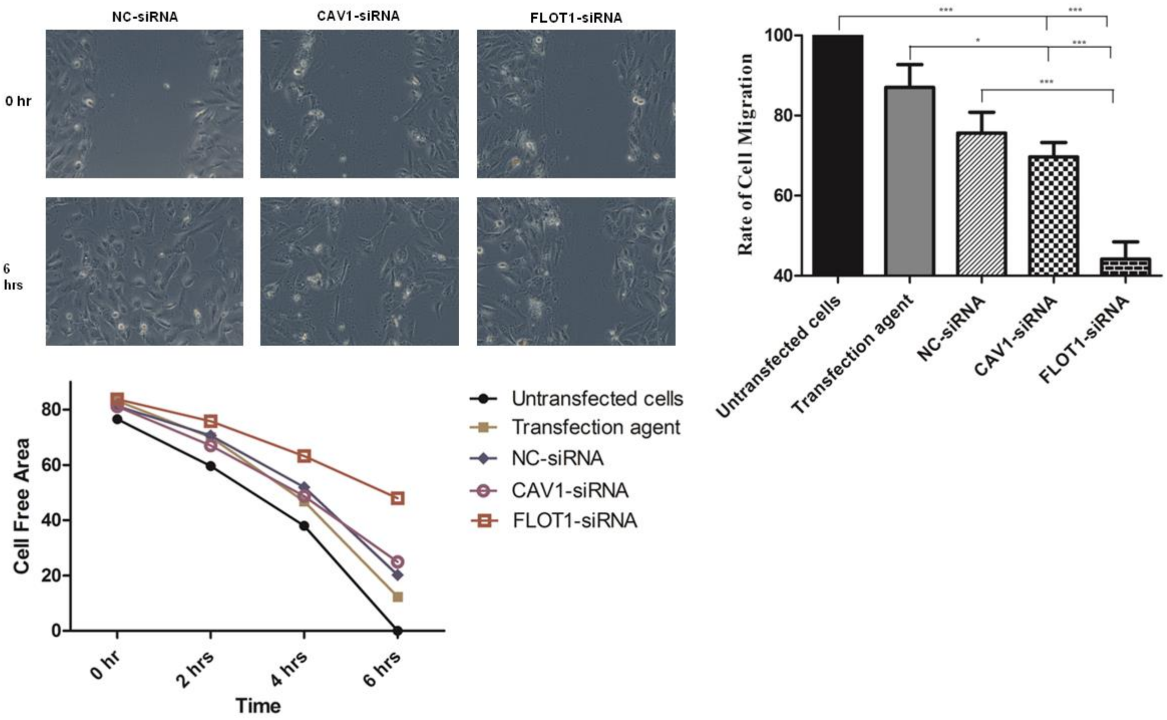

Supplementary Figure 5. Effect of CAV1 and FLOT1 silencing on cellular migration

Representative photomicrographs and bar graphs showing the effect knockdown of CAV1 and FLOT1 using specific siRNAs on the migration rate of the 786-O RCC cell line. The top row shows the cells at the time of wounding (0 h), and the bottom row shows cellular migration after 6 hours. CAV1-siRNA and FLOT1-siRNA significantly decreased the rate of cell migration, with incomplete wound closure after 6h, compared to controls.

Supplementary Table 1. Tissue specific miRNA-mRNA interactions filtered for the term "RCC associated genes"

TS target prediction	miRNA Name	MIMAT ID	Source	Confidence	Target Gene Symbol
1	miR-106b-5p	MIMAT0000680	TargetScan Human	High (predicted)	PDGFRA
2	miR-106b-5p	MIMAT0000680	TargetScan Human	High (predicted)	THRB
3	miR-10b-5p	MIMAT0000254	TargetScan Human	High (predicted)	FLT1
4	miR-10b-5p	MIMAT0000254	TargetScan Human	High (predicted)	INHBB
5	miR-124-3p	MIMAT0000422	TarBase, TargetScan Human, miRecords	Experimentally Observed, High (predicted)	CAV1
6	miR-124-3p	MIMAT0000422	TargetScan Human	High (predicted)	GNA13
7	miR-124-3p	MIMAT0000422	TargetScan Human	High (predicted)	SLC1A4
8	miR-133b	MIMAT0000770	TargetScan Human	High (predicted)	EGFR
9	miR-133b	MIMAT0000770	TargetScan Human	High (predicted)	FLT1
10	miR-136-5p	MIMAT0000448	TargetScan Human	High (predicted)	ADAM28
11	miR-136-5p	MIMAT0000448	TargetScan Human	High (predicted)	KDR
12	miR-138-5p	MIMAT0000430	TargetScan Human	High (predicted)	INHBB
13	miR-138-5p	MIMAT0000430	TargetScan Human	High (predicted)	KDR
14	miR-138-5p	MIMAT0000432	TargetScan Human	High (predicted)	GNA13
15	miR-149-5p	MIMAT0000450	TargetScan Human	High (predicted)	EGFR
16	miR-149-5p	MIMAT0000450	TargetScan Human	High (predicted)	FLT1
17	miR-15a-5p	MIMAT0000068	TarBase, TargetScan Human	Experimentally Observed, Moderate (predicted)	PNP
18	miR-15a-5p	MIMAT0000068	TargetScan Human	High (predicted)	THRB
19	miR-15a-5p	MIMAT0000068	miRecords	Experimentally Observed	WT1
20	miR-185-5p	MIMAT0000455	TargetScan Human	High (predicted)	PNP
21	miR-18a-5p	MIMAT0000072	TargetScan Human	High (predicted)	FGFR3
22	miR-18a-5p	MIMAT0000072	TargetScan Human	High (predicted)	KIT
23	miR-199a-5p	MIMAT0000231	TargetScan Human	High (predicted)	CAV1
24	miR-199a-5p	MIMAT0000231	TargetScan Human	High (predicted)	TGFA
25	miR-199a-5p	MIMAT0000231	TargetScan Human	High (predicted)	VEGFA
26	miR-200c-3p	MIMAT0000617	TargetScan Human	High (predicted)	CDH6
27	miR-200c-3p	MIMAT0000617	TargetScan Human	High (predicted)	FLT1
28	miR-200c-3p	MIMAT0000617	TargetScan Human	High (predicted)	FN1
29	miR-200c-3p	MIMAT0000617	TargetScan Human	High (predicted)	KDR
30	miR-200c-3p	MIMAT0000617	TargetScan Human	High (predicted)	TIMP2
31	miR-200c-3p	MIMAT0000617	TargetScan Human	High (predicted)	TUBB
32	miR-200c-3p	MIMAT0000617	TargetScan Human	High (predicted)	VEGFA
33	miR-203a-5p	MIMAT0000264	TargetScan Human	High (predicted)	CAV1
34	miR-203a-5p	MIMAT0000264	TargetScan Human	High (predicted)	CDH6

35	miR-203a-5p	MIMAT0000264	TargetScan Human	High (predicted)	VEGFA
36	miR-204-5p	MIMAT0000265	miRecords	Experimentally Observed	MMP9
37	miR-214-3p	MIMAT0000271	TargetScan Human	High (predicted)	SERPINA1
38	miR-214-3p	MIMAT0000271	TargetScan Human	High (predicted)	TIMP2
39	miR-21-5p	MIMAT0000076	TargetScan Human	High (predicted)	THRB
40	miR-218-5p	MIMAT0000275	TargetScan Human	High (predicted)	INHBB
41	miR-224-5p	MIMAT0000281	TargetScan Human	High (predicted)	DIO1
42	miR-23b-3p	MIMAT0000418	TargetScan Human, miRecords	Experimentally Observed, High (predicted)	MET
43	miR-23b-3p	MIMAT0000418	TargetScan Human	High (predicted)	TGFA
44	miR-23b-3p	MIMAT0000418	TargetScan Human	High (predicted)	TOP2A
45	miR-30a-3p	MIMAT0000088	TarBase, miRecords	Experimentally Observed	TUBA1A
46	miR-30a-5p	MIMAT0000087	TargetScan Human	High (predicted)	GNA13
47	miR-30a-5p	MIMAT0000087	TargetScan Human	High (predicted)	IFNAR2
48	miR-30a-5p	MIMAT0000087	TarBase	Experimentally Observed	MET
49	miR-30a-5p	MIMAT0000087	TargetScan Human	High (predicted)	TIMP2
50	miR-335-5p	MIMAT0000765	TargetScan Human	High (predicted)	FLT1
51	miR-340-5p	MIMAT0004692	TargetScan Human	High (predicted)	KIT
52	miR-340-5p	MIMAT0004692	TargetScan Human	High (predicted)	PDGFRA
53	miR-34a-5p	MIMAT0000255	TargetScan Human	High (predicted)	KIT
54	miR-34a-5p	MIMAT0000255	TargetScan Human	High (predicted)	PDGFRA
55	miR-34a-5p	MIMAT0000255	TargetScan Human	High (predicted)	RAP1GAP
56	miR-377-3p	MIMAT0000730	TargetScan Human	High (predicted)	SOD2
57	miR-377-3p	MIMAT0000730	TargetScan Human	High (predicted)	VEGFA
58	miR-454-3p	MIMAT0003885	TargetScan Human	High (predicted)	KIT
59	miR-454-3p	MIMAT0003885	TargetScan Human	High (predicted)	PDGFRA
60	miR-9-5p	MIMAT0000441	TargetScan Human	High (predicted)	CSDA
61	miR-9-5p	MIMAT0000441	TargetScan Human	High (predicted)	FLT1
62	miR-9-5p	MIMAT0000441	TargetScan Human	High (predicted)	GNA13
63	miR-9-5p	MIMAT0000441	TargetScan Human	High (predicted)	INHBB

Supplementary Table 2. 35-node networks functional annotation

ID	Molecules in Network	Score	Focus Molecules	Top Functions
1	ADAM28, Akt, c-Src, CAV1, EGFR, ERK, ERK1/2, FGFR3, Fibrin, Fibrinogen, FLT1, FN1, Focal adhesion kinase, growth factor receptor, Hsp90, Integrin, KDR, KIT, Mapk, MET, miR-23a-3p (and other miRNAs w/seed UCACAUU), miR-34a-5p (and other miRNAs w/seed GGCAGUG), MMP9, Neuropilin, p85 (pik3r), PDGFRA, PI3K (complex), PLC gamma, PP2A, Shc, STAT5a/b, TGFA, Vegf, VEGFA, WT1	32	16	Cancer, Renal and Urological Disease, Respiratory Disease
2	2-methoxyestradiol, C1orf56, C2orf43, CDH6, CSDA, CST4, CTNNB1, ESR1, FAM122C, GDAP1, GNA13, HDHD2, HSDL2, INHBB, KDELC2, LRRC8C, miR-130a-3p (and other miRNAs w/seed AGUGCAA), miR-16-5p (and other miRNAs w/seed AGCAGCA), miR-30a-3p (and other miRNAs w/seed UUUCAGU), miR-30c-5p (and other miRNAs w/seed GUAAACA), NT5DC1, PANX1, PNKD, PNP, PPARA, RAP1GAP, RFT1, SERPINA1, SLC1A4, SLC38A5, TOP2A, TPPP3, TUBA1A, TUBB, UBC	31	15	Cancer, Renal and Urological Disease, Endocrine System Disorders
3	AHRR, ALDH1A2, Ap1, ATL3, CREBBP, DRAM1, E2F1, GAS2L1, GNL3L, IFNA2, IFNAR2, IL11, JAKMIP1, MICA, mir-149, mir-224, miR-124-3p (and other miRNAs w/seed AAGGCAC), miR-138-5p (miRNAs w/seed GCUGGUG), miR-149-5p (miRNAs w/seed CUGGCUC), miR-17-5p (and other miRNAs w/seed AAAGUGC), miR-18a-5p (and other miRNAs w/seed AAGGUGC), miR-204-5p (and other miRNAs w/seed UCCCUUU), miR-218-5p (and other miRNAs w/seed UGUGCUU), miR-224-5p (miRNAs w/seed AAGUCAC), miR-9-5p (and other miRNAs w/seed CUUUGGU), NFKB1, ONECUT2, PRDM2, SP1, STAT3, TERT, TIMP2, TNFSF9, TP53, UGDH	21	11	Cellular Development, Cellular Growth and Proliferation, Cancer
4	AKT1, ALPI, BTG3, DIO1, DKK1, DOT1L, ELMO2, FLT1, Histone h3, Histone h4, Hsp70, KLF4, mir-8, miR-10a-5p (and other miRNAs w/seed ACCCUGU), miR-141-3p (and other miRNAs w/seed AACACUG), miR-185-5p (and other miRNAs w/seed GGAGAGA), miR-199a-5p (and other miRNAs w/seed CCAGUGU), miR-200b-3p (and other miRNAs w/seed AAUACUG), miR-214-3p (and other miRNAs w/seed CAGCAGG), NKD1, OASL, OPN1LW, OPN1SW, PPFIA2, PPFIA3, PPM1F, PTEN, PTPN13, PTPRD, RNA polymerase II, SET, SOD2, THRB, WDR37	18	10	Cancer, Renal and Urological Disease, Endocrine System Disorders
5	mir-154,miR-377-3p (miRNAs w/seed UCACACA)	2	1	Cell Death and Survival, Developmental Disorder, Hereditary Disorder
6	mir-335,miR-335-5p (and other miRNAs w/seed CAAGAGC)	2	1	Hereditary Disorder, Skeletal and Muscular Disorders, Endocrine System Disorders
7	mir-340,miR-340-5p (miRNAs w/seed UAUAAAG)	2	1	Cancer, Hematological Disease, Reproductive System Disease
8	mir-136,miR-136-5p (miRNAs w/seed CUCCAUU),RTL1	2	1	Organ Development, Organ Morphology, Reproductive System Development and Function

Supplementary Table 3. Pathways altered by transfection of miR-124-3p, miR-200c-3p and miR-30a-5p mimics transfection

Ingenuity Canonical Pathways	-log(p)	Ratio	Molecules
Molecular Mechanisms of Cancer	4.11000	6.89E-02	NRAS,CASP3,PAK6,RRAS,FZD9,PSEN2,RAP1A,GNAI2,ADCY9,CCNE1,CTNNA2,RHOG,NLK,CCND3,TGFB1,ARHGEF16,PIK3R6,RBPJ,BID,LEF1,CDKN1B,BMP6,E2F2,CDC25A,CAMK2B
Integrin Signaling	3.30000	7.80E-02	MPRIP,NRAS,PAK6,RRAS,PPP1CB,TSPAN2,TLN1,ITGB8,RAP1A,RHOG,CAPNS1,PLCG2,CAV1,PIK3R6,ITGA1,ITGB4
Isoleucine Degradation I	3.10000	2.86E-01	HADHB,ACADSB,SDS,HADHA
Estrogen-mediated S-phase Entry	3.08000	1.85E-01	CCNE1,CDKN1B,E2F2,SKP2,CDC25A
Cell Cycle: G1/S Checkpoint Regulation	3.08000	1.18E-01	CCNE1,CCND3,TGFB1,HDAC7,CDKN1B,E2F2,SKP2,CDC25A
Chemokine Signaling	2.86000	1.18E-01	GNAI2,MPRIP,NRAS,CCL4,RRAS,PLCG2,PPP1CB,CAMK2B
Regulation of the Epithelial-Mesenchymal Transition Pathway	2.74000	7.45E-02	MAML1,MET,WNT3A,NRAS,ARAF,TGFB1,RRAS,PIK3R6,RBPJ,LEF1,PSEN2,FZD9,FGFRL1,CLDN3
Fatty Acid β-oxidation I	2.69000	1.72E-01	HADHB,ACSL3,ECI2,SDS,HADHA
Virus Entry via Endocytic Pathways	2.68000	9.78E-02	NRAS,RRAS,PLCG2,PIK3R6,CAV1,ITGA1,ITGB4,ITGB8,CXADR
Valine Degradation I	2.66000	2.22E-01	HADHB,ACADSB,SDS,HADHA
Glioblastoma Multiforme Signaling	2.63000	7.64E-02	PLCD3,CCNE1,RHOG,NRAS,WNT3A,RRAS,PLCG2,PIK3R6,FZD9,LEF1,CDKN1B,E2F2
Tryptophan Degradation III (Eukaryotic)	2.57000	2.11E-01	HADHB,L3HYPDH,HADHA,KYNU
Cyclins and Cell Cycle Regulation	2.51000	9.20E-02	CCNE1,CCND3,TGFB1,HDAC7,CDKN1B,E2F2,SKP2,CDC25A
Breast Cancer Regulation by Stathmin1	2.50000	7.11E-02	NRAS,RRAS,PPP1CB,GNG10,GNAI2,ADCY9,STMN1,CCNE1,TUBB6,ARHGEF16,PIK3R6,CDKN1B,E2F2,CAMK2B
Ketogenesis	2.49000	3.00E-01	HADHB,HMGCS1,HADHA
D-myo-inositol-5-phosphate Metabolism	2.45000	7.48E-02	PLCD3,PTPRO,PLCG2,PPM1H,PTPN1,PPM1F,ALPL,PTPN12,THTPA,PTPN22,CDC25A
Synaptic Long Term Potentiation	2.40000	8.55E-02	PLCD3,NRAS,PPP3CB,RRAS,GRM1,GRM3,PLCG2,PPP1CB,RAP1A,CAMK2B
Role of NFAT in Cardiac Hypertrophy	2.37000	6.95E-02	NRAS,RRAS,GNG10,GNAI2,PLCD3,ADCY9,PPP3CB,TGFB1,PLCG2,HDAC7,PIK3R6,RCAN3,CAMK2B
Paxillin Signaling	2.36000	8.18E-02	NRAS,PAK6,RRAS,PIK3R6,TLN1,ITGA1,ITGB4,ITGB8,PTPN12
Glutaryl-CoA Degradation	2.36000	2.73E-01	HADHB,L3HYPDH,HADHA
Mevalonate Pathway I	2.24000	2.50E-01	HADHB,HMGCS1,HADHA
Macropinocytosis Signaling	2.23000	9.21E-02	MET,NRAS,RRAS,PLCG2,PIK3R6,ITGB4,ITGB8
Renal Cell Carcinoma Signaling	2.20000	9.86E-02	MET,NRAS,PAK6,RRAS,TGFB1,PIK3R6,RAP1A
Thrombin Signaling	2.15000	6.60E-02	MPRIP,NRAS,RRAS,PPP1CB,GNG10,GNAI2,PLCD3,ADCY9,RHOG,PLCG2,ARHGEF16,PIK3R6,CAMK2B
Regulation of Cellular Mechanics by Calpain Protease	2.10000	8.57E-02	CCNE1,NRAS,CAPNS1,RRAS,TLN1,CDKN1B

Thyroid Cancer Signaling	2.07000	1.22E-01	NRAS,GDNF,BDNF,RRAS,LEF1
Antiproliferative Role of TOB in T Cell Signaling	2.06000	1.54E-01	CCNE1,TGFB1,CDKN1B,SKP2
Chronic Myeloid Leukemia Signaling	2.04000	7.84E-02	NRAS,RRAS,TGFB1,CTBP2,HDAC7,PIK3R6,CDKN1B,E2F2
Acetate Conversion to Acetyl-CoA	2.02000	4.00E-01	ACSL3,ACSS1
3-phosphoinositide Degradation	1.99000	6.80E-02	PTPRO,PPM1H,PTPMT1,PTPN1,PPM1F,ALPL,PTPN12,THTPA,PTPN22,CDC25A
HER-2 Signaling in Breast Cancer	1.98000	8.75E-02	CCNE1,NRAS,RRAS,PIK3R6,ITGB4,ITGB8,CDKN1B
Oxidative Ethanol Degradation III	1.96000	1.11E-01	ACSL3,ACSS1,ALDH9A1
D-myo-inositol (1,4,5,6)-Tetrakisphosphate Biosynthesis	1.92000	6.92E-02	PTPRO,PPM1H,PTPN1,PPM1F,ALPL,PTPN12,THTPA,PTPN22,CDC25A
D-myo-inositol (3,4,5,6)-tetrakisphosphate Biosynthesis	1.92000	6.92E-02	PTPRO,PPM1H,PTPN1,PPM1F,ALPL,PTPN12,THTPA,PTPN22,CDC25A
Superpathway of Geranylgeranyldiphosphate Biosynthesis I (via Mevalonate)	1.88000	1.88E-01	HADHB,HMGCS1,HADHA
Superpathway of Inositol Phosphate Compounds	1.87000	6.09E-02	PLCD3,PTPRO,PLCG2,PPM1H,PTPN1,PIK3R6,PPM1F,ALPL,PTPN12,THTPA,PTPN22,CDC25A

Protein Kinase A Signaling	1.86000	5.36E-02	PPP1CB,AKAP6,PTPN12,RAP1A,GNG10,GNAI2,ADCY9,PLCD3,ADD3,H3F3A/H3F3B,PDE7B,PPP3CB,TGFB1,PTPRO,PLCG2,PTPN1,LEF1,PTPN22,CDC25A,CAMK2B
Gαi Signaling	1.83000	7.03E-02	GNAI2,S1PR3,ADCY9,NRAS,RRAS,GRM3,CAV1,RAP1A,GNG10
Phosphatidylglycerol Biosynthesis II (Non-plastidic)	1.80000	1.76E-01	LCLAT1,CDS1,PTPMT1
Ethanol Degradation IV	1.80000	1.76E-01	ACSL3,ACSS1,ALDH9A1
3-phosphoinositide Biosynthesis	1.79000	6.29E-02	PTPRO,PPM1H,PTPN1,PIK3R6,PPM1F,ALPL,PTPN12,THTPA,PTPN22,CDC25A
Endoplasmic Reticulum Stress Pathway	1.73000	1.67E-01	CASP3,MBTPS1,TAOK3
CREB Signaling in Neurons	1.70000	5.95E-02	GNAI2,PLCD3,ADCY9,NRAS,RRAS,GRM1,GRM3,PLCG2,PIK3R6,CAMK2B,GNG10
Agrin Interactions at Neuromuscular Junction	1.70000	8.96E-02	LAMC1,NRAS,PAK6,RRAS,LAMA2,ITGA1
FAK Signaling	1.70000	7.14E-02	NRAS,CAPNS1,PAK6,RRAS,PLCG2,PIK3R6,TLN1
GDNF Family Ligand-Receptor Interactions	1.68000	8.57E-02	NRAS,ARTN,GDNF,RRAS,PLCG2,PIK3R6
Sphingosine-1-phosphate Signaling	1.65000	6.96E-02	GNAI2,S1PR3,PLCD3,ADCY9,RHOG,CASP3,PLCG2,PIK3R6
Gap Junction Signaling	1.64000	6.41E-02	GNAI2,PLCD3,ADCY9,NRAS,TUBB6,PPP3CB,RRAS,PLCG2,PIK3R6,CAV1
Endometrial Cancer Signaling	1.61000	9.09E-02	CTNNA2,NRAS,RRAS,PIK3R6,LEF1

Ketolysis	1.60000	2.50E-01	HADHB,HADHA
Human Embryonic Stem Cell Pluripotency	1.59000	6.04E-02	S1PR3,WNT3A,BDNF,TGFB1,PIK3R6,FZD9,LEF1,FGFRL1,BMP6
Aryl Hydrocarbon Receptor Signaling	1.57000	6.38E-02	TRIP11,CCNE1,NFIC,ALDH1L2,CCND3,TGFB1,CDKN1B,ALDH3B2,ALDH9A1
Dopamine Degradation	1.55000	1.43E-01	SULT1A1,SULT1C3,ALDH9A1
CCR3 Signaling in Eosinophils	1.55000	6.61E-02	GNAI2,MPRIP,NRAS,PAK6,RRAS,PIK3R6,PPP1CB,GNG10
Notch Signaling	1.53000	9.52E-02	MAML1,DLL1,RBPJ,PSEN2
Phospholipase C Signaling	1.52000	5.33E-02	ADCY9,RHOG,NRAS,MPRIP,PPP3CB,CD3E,RRAS,ARHGEF16,PLCG2,HDAC7,PPP1CB ,RAP1A,GNG10
Heme Biosynthesis II	1.50000	2.22E-01	CPOX,HMBS
Threonine Degradation II	1.50000	1.00E+00	GCAT
14-3-3-mediated Signaling	1.49000	6.78E-02	PLCD3,NRAS,TUBB6,RRAS,PLCG2,PIK3R6,VIM,CDKN1B
P2Y Purigenic Receptor Signaling Pathway	1.45000	6.40E-02	GNAI2,PLCD3,ADCY9,NRAS,RRAS,PLCG2,PIK3R6,GNG10
Acute Myeloid Leukemia Signaling	1.44000	7.41E-02	NRAS,ARAF,RRAS,PIK3R6,CEBPA,LEF1
Myc Mediated Apoptosis Signaling	1.43000	8.33E-02	NRAS,CASP3,RRAS,PIK3R6,BID
ErbB4 Signaling	1.43000	8.06E-02	NRAS,RRAS,PLCG2,PIK3R6,PSEN2
Regulation of IL-2 Expression in Activated and Anergic T Lymphocytes	1.42000	7.14E-02	NRAS,PPP3CB,CD3E,RRAS,TGFB1,PLCG2

Neuropathic Pain Signaling In Dorsal Horn Neurons	1.41000	6.86E-02	PLCD3,BDNF,GRM1,GRM3,PLCG2,PIK3R6,CAMK2B
FcγRIIB Signaling in B Lymphocytes	1.39000	8.16E-02	NRAS,RRAS,PLCG2,PIK3R6
Epithelial Adherens Junction Signaling	1.38000	6.25E-02	MET,DLL1,CTNNA2,NRAS,TUBB6,RRAS,LEF1,RAP1A,ACVR1B
Antiproliferative Role of Somatostatin Receptor 2	1.38000	7.81E-02	NRAS,RRAS,PIK3R6,CDKN1B,GNG10
Prostate Cancer Signaling	1.37000	6.59E-02	CCNE1,NRAS,RRAS,PIK3R6,LEF1,CDKN1B
Role of Macrophages, Fibroblasts and Endothelial Cells in Rheumatoid Arthritis	1.36000	4.82E-02	NRAS,RRAS,FZD9,IL1R2,PLCD3,WNT3A,NLK,PPP3CB,F2RL1,TGFB1,PLCG2,CEBPA, PIK3R6,LEF1,CAMK2B
cAMP-mediated signaling	1.35000	5.50E-02	GNAI2,S1PR3,ADCY9,PDE7B,PPP3CB,GRM3,HTR7,AKAP6,PKIA,ADORA2A,RAP1A,C AMK2B
GM-CSF Signaling	1.32000	7.58E-02	NRAS,PPP3CB,RRAS,PIK3R6,CAMK2B
Cardiac Hypertrophy Signaling	1.31000	5.24E-02	GNAI2,PLCD3,ADCY9,HAND2,RHOG,NRAS,PPP3CB,RRAS,TGFB1,PLCG2,PIK3R6,GN G10
Estrogen Receptor Signaling	1.31000	6.02E-02	NRAS,H3F3A/H3F3B,CCNC,MED20,RRAS,CTBP2,NR3C1,MED4

Supplementary Table 4. (**A**) Pathway analysis of miR-30a-5p effect evaluated by microarray in ccRCC cells; (**B**) Gene ontology analysis of miR-30a-5p effect on gene expression

4A: Pathway analysis of miR-30a-5p effect evaluated by microarray in ccRCC cells

**Ingenuity Canonical Pathways**	**-log(p)**	**Ratio**	**Molecules**
Heparan Sulfate Biosynthesis (Late Stages)	2.55	0.106	SULT1A1,SULT1C2,SULT1A3/SULT1A4,GLCE,EXTL2
Phosphatidylglycerol Biosynthesis II (Non-plastidic)	2.31	0.176	LCLAT1,CDS1,PTPMT1
Heparan Sulfate Biosynthesis	2.29	0.0926	SULT1A1,SULT1C2,SULT1A3/SULT1A4,GLCE,EXTL2
Endoplasmic Reticulum Stress Pathway	2.24	0.167	CASP3,MBTPS1,TAOK3
Notch Signaling	2.15	0.0952	MAML1,RBPJ,PSEN2,JAG1
DNA Methylation and Transcriptional Repression Signaling	2.11	0.13	DNMT3B,DNMT3A,SAP30
Protein Kinase A Signaling	2.02	0.0403	PTPRE,YWHAZ,PPP1CB,AKAP6,RAP1A,GNG10,GNAI2,PLCD3,GNB3,H3F3A/H3F3B,PLCG2,EYA3,PTPN1,KDELR3,PTPN22
Cell Cycle: G1/S Checkpoint Regulation	2.01	0.0735	CCNE1,CCND3,HDAC7,E2F2,SKP2
GÎ±i Signaling	1.92	0.0551	GNAI2,S1PR3,GNB3,NRAS,GRM3,RAP1A,GNG10
LPS/IL-1 Mediated Inhibition of RXR Function	1.92	0.0452	IL1R2,ALDH1L2,SULT1A1,SULT1C2,SULT1A3/SULT1A4,FABP5,GSTO2,ALDH3B2,HMGCS1,PAPSS2
CCR5 Signaling in Macrophages	1.9	0.0617	GNAI2,GNB3,CD3E,PLCG2,GNG10
Estrogen-mediated S-phase Entry	1.89	0.111	CCNE1,E2F2,SKP2
Breast Cancer Regulation by Stathmin1	1.75	0.0457	GNAI2,STMN1,CCNE1,GNB3,NRAS,PPP1CB,TUBB4A,E2F2,GNG10
D-myo-inositol (1,4,5)-Trisphosphate Biosynthesis	1.75	0.111	PLCD3,PLCG2,PLCH1
Amyloid Processing	1.7	0.0755	CAPNS1,BACE1,PSEN2,APP
S-methyl-5'-thioadenosine Degradation II	1.68	1	MTAP
Cyclins and Cell Cycle Regulation	1.66	0.0575	CCNE1,CCND3,HDAC7,E2F2,SKP2
Molecular Mechanisms of Cancer	1.64	0.0358	GNAI2,CCNE1,CTNNA2,NLK,NRAS,CCND3,CASP3,RBPJ,PSEN2,HIF1A,BMP6,RAP1A,E2F2
Role of NFAT in Regulation of the Immune Response	1.63	0.043	GNAI2,RCAN1,GNB3,NRAS,CD3E,PLCG2,IKBKAP,GNG10
Sphingosine-1-phosphate Signaling	1.6	0.0522	GNAI2,S1PR3,PLCD3,CASP3,PLCG2,CASP2
GÎ±s Signaling	1.6	0.0522	GNB3,HTR7,MC1R,ADORA2A,RAP1A,GNG10
G Protein Signaling Mediated by Tubby	1.59	0.0811	GNB3,PLCG2,GNG10
Phospholipases	1.57	0.0727	PLCD3,PLCG2,PLA1A,PLCH1
Glutamate Receptor Signaling	1.57	0.0667	GNB3,SLC1A4,GRM3,SLC1A1
Complement System	1.55	0.0909	C5AR1,C7,C1QC
Role of NFAT in Cardiac Hypertrophy	1.51	0.0428	GNAI2,PLCD3,RCAN1,GNB3,NRAS,PLCG2,HDAC7,GNG10
Î±-Adrenergic Signaling	1.5	0.0543	GNAI2,GNB3,NRAS,PLCG2,GNG10
Regulation of the Epithelial-Mesenchymal Transition Pathway	1.49	0.0428	MET,MAML1,NRAS,RBPJ,PSEN2,HIF1A,JAG1,CLDN3
Synaptic Long Term Potentiation	1.49	0.0517	PLCD3,NRAS,GRM3,PLCG2,PPP1CB,RAP1A
Vitamin-C Transport	1.48	0.143	GSTO2,SLC2A3
14-3-3-mediated Signaling	1.47	0.0508	PLCD3,NRAS,PLCG2,YWHAZ,VIM,TUBB4A
Role of BRCA1 in DNA Damage Response	1.47	0.0645	SMARCD2,SLC19A1,RFC1,E2F2

Cell Cycle Regulation by BTG Family Proteins	1.45	0.0833	CCNE1,CNOT7,E2F2
G Beta Gamma Signaling	1.44	0.05	GNAI2,GNB3,NRAS,PLCG2,GNG10
Apoptosis Signaling	1.44	0.0543	NRAS,CAPNS1,CASP3,PLCG2,CASP2
P2Y Purigenic Receptor Signaling Pathway	1.44	0.048	GNAI2,PLCD3,GNB3,NRAS,PLCG2,GNG10
CDP-diacylglycerol Biosynthesis I	1.42	0.133	LCLAT1,CDS1
Flavin Biosynthesis IV (Mammalian)	1.39	0.5	RFK
Sulfate Activation for Sulfonation	1.39	0.5	PAPSS2
Cardiolipin Biosynthesis II	1.39	0.5	PTPMT1
Mismatch Repair in Eukaryotes	1.37	0.1	SLC19A1,RFC1
Estrogen Receptor Signaling	1.32	0.0451	NRAS,H3F3A/H3F3B,CCNC,MED20,TAF5L,MED4
Dermatan Sulfate Biosynthesis (Late Stages)	1.3	0.075	SULT1A1,SULT1C2,SULT1A3/SULT1A4
**4B: Gene ontology analysis of miR-30a-5p effect on gene expression**			
**Description**	**Term_ID**	**log10 p-value**	
negative regulation of transcription from RNA polymerase II promoter	GO:0000122	-8.3089	
positive regulation of transcription from RNA polymerase II promoter	GO:0045944	-7.9172	
transcription, DNA-dependent	GO:0006351	-7.2457	
transcription initiation from RNA polymerase II promoter	GO:0006367	-5.5129	
negative regulation of transcription, DNA-dependent	GO:0045892	-5.1618	
transforming growth factor beta receptor signaling pathway	GO:0007179	-4.9031	
protein ubiquitination involved in ubiquitin-dependent protein catabolic process	GO:0042787	-4.6778	

negative regulation of neuron apoptotic process	GO:0043524	-4.6234	
small GTPase mediated signal transduction	GO:0007264	-4.5243	
transcription from RNA polymerase II promoter	GO:0006366	-4.4672	
prostate gland epithelium morphogenesis	GO:0060740	-4.4112	
insulin-like growth factor receptor signaling pathway	GO:0048009	-4.4023	
in utero embryonic development	GO:0001701	-4.3233	
positive regulation of transcription, DNA-dependent	GO:0045893	-4.279	
cell communication	GO:0007154	-4.2725	
protein ubiquitination	GO:0016567	-4.1355	
protein phosphorylation	GO:0006468	-4.0414	
protein O-linked glycosylation via threonine	GO:0018243	-3.8597	
protein O-linked glycosylation via serine	GO:0018242	-3.8597	
ER to Golgi vesicle-mediated transport	GO:0006888	-3.617	
T cell differentiation	GO:0030217	-3.5712	
actomyosin structure organization	GO:0031032	-3.4772	
intracellular protein transport	GO:0006886	-3.4595	
maternal placenta development	GO:0001893	-3.3751	
N-acetylglucosamine metabolic process	GO:0006044	-3.3042	
NLS-bearing substrate import into nucleus	GO:0006607	-3.3042	
protein K48-linked ubiquitination	GO:0070936	-3.298	
mammary gland development	GO:0030879	-3.2807	
cochlea morphogenesis	GO:0090103	-3.2807	

posttranscriptional regulation of gene expression	GO:0010608	-3.2748	
COPII vesicle coating	GO:0048208	-3.1469	
histone H3-K4 methylation	GO:0051568	-3.1469	
negative regulation of apoptotic process	GO:0043066	-3.0578	
protein N-linked glycosylation via asparagine	GO:0018279	-3.0556	
regulation of neuron differentiation	GO:0045664	-3.003	
cell cycle	GO:0007049	-2.9937	
T cell activation	GO:0042110	-2.984	
proteasomal ubiquitin-dependent protein catabolic process	GO:0043161	-2.9713	
positive regulation of macroautophagy	GO:0016239	-2.9342	
negative regulation of myoblast differentiation	GO:0045662	-2.9342	
thymus development	GO:0048538	-2.896	
dichotomous subdivision of terminal units involved in salivary gland branching	GO:0060666	-2.894	
fear response	GO:0042596	-2.894	
inositol metabolic process	GO:0006020	-2.894	
Golgi organization	GO:0007030	-2.8155	
protein complex assembly	GO:0006461	-2.7563	
positive regulation of smooth muscle cell migration	GO:0014911	-2.756	
trophoblast giant cell differentiation	GO:0060707	-2.756	
post-translational protein modification	GO:0043687	-2.7448	
positive regulation of B cell proliferation	GO:0030890	-2.7381	

Rho protein signal transduction	GO:0007266	-2.7099	
negative regulation of cell proliferation	GO:0008285	-2.6688	
activation of MAPKK activity	GO:0000186	-2.6467	
regulation of actin polymerization or depolymerization	GO:0008064	-2.61	
lens induction in camera-type eye	GO:0060235	-2.61	
cell-substrate junction assembly	GO:0007044	-2.61	
sympathetic nervous system development	GO:0048485	-2.5737	
cochlear nucleus development	GO:0021747	-2.5727	
negative regulation of histone methylation	GO:0031061	-2.5727	
regulation of response to stress	GO:0080134	-2.5727	
regulation of transcription involved in S phase of mitotic cell cycle	GO:0000115	-2.5727	
tube development	GO:0035295	-2.5727	
atrioventricular node development	GO:0003162	-2.5727	
regulation of protein tyrosine kinase activity	GO:0061097	-2.5727	
auditory receptor cell fate commitment	GO:0009912	-2.5727	
heart development	GO:0007507	-2.554	
negative regulation of Ras protein signal transduction	GO:0046580	-2.5277	
peptidyl-threonine phosphorylation	GO:0018107	-2.4922	
embryonic hindlimb morphogenesis	GO:0035116	-2.4922	

Supplementary Table 5. (**A**) Pathway analysis of miR-124-3p effect evaluated by microarray in ccRCC cells; (**B**) Gene ontology analysis of miR-124-3p effect on gene expression

**5A: Pathway analysis of miR-124-3p effect evaluated by microarray in ccRCC cells**			
**Ingenuity Canonical Pathways**	**-log(p)**	**Ratio**	**Molecules**
Regulation of Cellular Mechanics by Calpain Protease	6.42E+00	1.57E-01	ITGB1,CCNE1,RRAS2,CAPNS1,GRB2,RRAS,CDK6,CDK4,TLN1,CAPN2,CDKN1B
Cell Cycle: G1/S Checkpoint Regulation	4.94E+00	1.47E-01	CDKN2D,CCNE1,CCND3,TGFB1,HDAC7,CDK6,CDK4,CDKN1B,RBL1,SKP2
Fatty Acid β-oxidation I	3.83E+00	2.07E-01	HADHB,ACSL3,ECI2,ACAA2,HADHA,HADH
Glutaryl-CoA Degradation	3.72E+00	3.64E-01	HADHB,L3HYPDH,HADHA,HADH
Molecular Mechanisms of Cancer	3.54E+00	6.06E-02	MAP2K6,GRB2,RRAS,GNA12,CDK6,CDK4,RBL1,RAP1A,ADCY9,CCNE1,CTNNA2,CDKN2D,RRAS2,RHOG,NLK,CCND3,TGFB1,ARHGEF16,MAP2K3,CDKN1B,BMP6,BCL2L11
HER-2 Signaling in Breast Cancer	3.49E+00	1.12E-01	ITGB1,CCNE1,RRAS2,GRB2,RRAS,CDK6,ITGB8,CDKN1B,ITGB7
Cyclins and Cell Cycle Regulation	3.45E+00	1.03E-01	CDKN2D,CCNE1,CCND3,TGFB1,HDAC7,CDK6,CDK4,CDKN1B,SKP2
Estrogen-mediated S-phase Entry	3.28E+00	1.85E-01	CCNE1,CDK4,CDKN1B,RBL1,SKP2
Antiproliferative Role of TOB in T Cell Signaling	3.11E+00	1.92E-01	CCNE1,TGFB1,CDKN1B,TWSG1,SKP2
Phosphatidylglycerol Biosynthesis II (Non-plastidic)	2.92E+00	2.35E-01	ABHD5,LCLAT1,CDS1,PTPMT1
Chronic Myeloid Leukemia Signaling	2.88E+00	8.82E-02	RRAS2,GRB2,RRAS,TGFB1,HDAC7,CDK6,CDK4,CDKN1B,RBL1
Integrin Signaling	2.83E+00	6.83E-02	ITGB1,GRB2,RRAS,PPP1CB,TLN1,ITGB8,RAP1A,ITGB7,RHOG,RRAS2,CAPNS1,CAV1,CAPN2,TSPAN6
Aryl Hydrocarbon Receptor Signaling	2.80E+00	7.80E-02	TRIP11,CCNE1,NFIC,ALDH1L2,CCND3,TGFB1,CDK6,CDK4,CDKN1B,ALDH3B2,ALDH9A1
Role of NFAT in Cardiac Hypertrophy	2.75E+00	6.95E-02	CTF1,MAP2K6,ADCY9,RCAN1,PLCD3,RRAS2,PPP3CB,TGFB1,RRAS,GRB2,HDAC7,MAP2K3,GNG10
Tryptophan Degradation III (Eukaryotic)	2.73E+00	2.11E-01	HADHB,L3HYPDH,HADHA,HADH
Gαi Signaling	2.64E+00	7.87E-02	S1PR3,ADCY9,RRAS2,GRB2,RRAS,GRM3,CAV1,GABBR1,RAP1A,GNG10
Ketogenesis	2.61E+00	3.00E-01	HADHB,HMGCS1,HADHA

Macropinocytosis Signaling	2.48E+00	9.21E-02	ITGB1,MET,RRAS2,RRAS,ITGB8,RAB34,ITGB7
TGF-β Signaling	2.45E+00	8.60E-02	MAP2K6,ZNF423,RRAS2,GRB2,RRAS,TGFB1,MAP2K3,ACVR1B
Mevalonate Pathway I	2.37E+00	2.50E-01	HADHB,HMGCS1,HADHA
Sertoli Cell-Sertoli Cell Junction Signaling	2.36E+00	6.56E-02	ITGB1,F11R,CTNNA2,RRAS2,TJP2,TUBB6,RRAS,SORBS1,CGN,ZAK,TUBB4A,MAP2K3
Heme Biosynthesis from Uroporphyrinogen-III I	2.32E+00	5.00E-01	FECH,CPOX
Germ Cell-Sertoli Cell Junction Signaling	2.30E+00	7.01E-02	ITGB1,MAP2K6,CTNNA2,RHOG,RRAS2,TUBB6,RRAS,TGFB1,SORBS1,TUBB4A,MAP2K3
Actin Nucleation by ARP-WASP Complex	2.28E+00	9.09E-02	ITGB1,RHOG,RRAS2,GRB2,RRAS,GNA12
Regulation of the Epithelial-Mesenchymal Transition Pathway	2.26E+00	6.42E-02	MET,MAP2K6,MAML1,RRAS2,ARAF,GRB2,RRAS,TGFB1,FGFR4,MAP2K3,BCL9,FGFRL1
Actin Cytoskeleton Signaling	2.12E+00	5.68E-02	ITGB1,TIAM1,CYFIP2,RRAS2,RRAS,GRB2,GNA12,MYH9,PPP1CB,PIP4K2B,TLN1,WASF2,PIP4K2A
Acetate Conversion to Acetyl-CoA	2.11E+00	4.00E-01	ACSL3,ACSS1
CDP-diacylglycerol Biosynthesis I	2.08E+00	2.00E-01	ABHD5,LCLAT1,CDS1
Oxidative Ethanol Degradation III	2.08E+00	1.11E-01	ACSL3,ACSS1,ALDH9A1
Epithelial Adherens Junction Signaling	2.06E+00	6.94E-02	MET,CTNNA2,RRAS2,TUBB6,RRAS,SORBS1,MYH9,TUBB4A,RAP1A,ACVR1B
Breast Cancer Regulation by Stathmin1	2.05E+00	6.09E-02	ADCY9,STMN1,CCNE1,RRAS2,TUBB6,GRB2,RRAS,ARHGEF16,PPP1CB,TUBB4A,CDKN1B,GNG10
Superpathway of Geranylgeranyldiphosphate Biosynthesis I (via Mevalonate)	2.00E+00	1.88E-01	HADHB,HMGCS1,HADHA
Ethanol Degradation II	1.99E+00	1.33E-01	ACSL3,ACSS1,PECR,ALDH9A1
Cardiac Hypertrophy Signaling	1.96E+00	5.68E-02	MAP2K6,ADCY9,PLCD3,RRAS2,RHOG,PPP3CB,TGFB1,RRAS,GRB2,GNA12,IL6R,MAP2K3,GNG10
FAK Signaling	1.93E+00	7.14E-02	ITGB1,RRAS2,CAPNS1,GRB2,RRAS,TLN1,CAPN2
Ethanol Degradation IV	1.92E+00	1.76E-01	ACSL3,ACSS1,ALDH9A1
Agrin Interactions at Neuromuscular Junction	1.90E+00	8.96E-02	ITGB1,LAMC1,RRAS2,RRAS,LAMA2,GABPA
Neurotrophin/TRK Signaling	1.90E+00	8.57E-02	MAP2K6,RRAS2,BDNF,GRB2,RRAS,MAP2K3
CDK5 Signaling	1.90E+00	7.87E-02	ITGB1,LAMC1,ADCY9,RRAS2,BDNF,RRAS,PPP1CB
Renal Cell Carcinoma Signaling	1.85E+00	8.45E-02	MET,RRAS2,GRB2,RRAS,TGFB1,RAP1A

GADD45 Signaling	1.79E+00	1.43E-01	CCNE1,CCND3,CDK4
Semaphorin Signaling in Neurons	1.78E+00	9.62E-02	ITGB1,MET,RHOG,DPYSL3,NRP1
Glioma Signaling	1.73E+00	6.60E-02	CDKN2D,RRAS2,GRB2,RRAS,CDK6,CDK4,RBL1
14-3-3-mediated Signaling	1.73E+00	6.78E-02	PLCD3,RRAS2,TUBB6,RRAS,GRB2,VIM,TUBB4A,CDKN1B
PTEN Signaling	1.71E+00	6.15E-02	ITGB1,RRAS2,GRB2,RRAS,FGFR4,FGFRL1,CDKN1B,BCL2L11
Ketolysis	1.69E+00	2.50E-01	HADHB,HADHA
Acute Myeloid Leukemia Signaling	1.63E+00	7.41E-02	MAP2K6,RRAS2,ARAF,GRB2,RRAS,MAP2K3
Paxillin Signaling	1.62E+00	6.36E-02	ITGB1,RRAS2,GRB2,RRAS,TLN1,ITGB8,ITGB7
Glioblastoma Multiforme Signaling	1.61E+00	5.73E-02	PLCD3,CCNE1,RHOG,RRAS2,GRB2,RRAS,CDK6,CDK4,CDKN1B
Heme Biosynthesis II	1.59E+00	2.22E-01	FECH,CPOX
RhoGDI Signaling	1.58E+00	5.29E-02	ITGB1,RHOG,GNA12,ARHGEF16,PIP4K2B,ARHGDIA,WASF2,CDH16,PIP4K2A,GNG10
Cholecystokinin/Gastrin-mediated Signaling	1.58E+00	6.93E-02	MAP2K6,RHOG,RRAS2,GRB2,RRAS,GNA12,MAP2K3
Ephrin Receptor Signaling	1.57E+00	5.08E-02	ITGB1,EPHA6,RRAS2,GRB2,RRAS,SORBS1,GNA12,RAP1A,EPHA2,GNG10
S-methyl-5'-thioadenosine Degradation II	1.54E+00	1.00E+00	MTAP
Sorbitol Degradation I	1.54E+00	1.00E+00	SORD
Rac Signaling	1.54E+00	5.98E-02	ITGB1,TIAM1,RRAS2,CYFIP2,RRAS,PIP4K2B,PIP4K2A
Gap Junction Signaling	1.51E+00	5.77E-02	PLCD3,ADCY9,RRAS2,TUBB6,PPP3CB,GRB2,RRAS,CAV1,TUBB4A
Phospholipase C Signaling	1.50E+00	4.92E-02	ITGB1,ADCY9,RHOG,RRAS2,PPP3CB,GRB2,RRAS,ARHGEF16,HDAC7,PPP1CB,RAP1A,GNG10
Pyridoxal 5'-phosphate Salvage Pathway	1.49E+00	8.06E-02	MAP2K6,ARAF,CDK6,CDK4,MAP2K3
FGF Signaling	1.47E+00	6.98E-02	MET,MAP2K6,GRB2,FGFR4,MAP2K3,FGFRL1
Sphingosine-1-phosphate Signaling	1.44E+00	6.09E-02	S1PR3,PLCD3,ADCY9,RHOG,GNA12,CASP2,SPHK1
Gαs Signaling	1.44E+00	6.09E-02	ADCY9,ADD3,HTR7,MC1R,ADORA2A,RAP1A,GNG10
Clathrin-mediated Endocytosis Signaling	1.42E+00	5.26E-02	ITGB1,MET,CD2AP,PPP3CB,GRB2,PICALM,ITGB8,ITGB7,RAB4B,HIP1R
D-myo-inositol-5-phosphate Metabolism	1.42E+00	5.44E-02	MTMR6,PLCD3,ATP1A1,PPM1H,PTPN1,PPM1F,PIP4K2A,PTPN22
Non-Small Cell Lung Cancer Signaling	1.41E+00	6.85E-02	RRAS2,GRB2,RRAS,CDK6,CDK4
UVA-Induced MAPK Signaling	1.41E+00	6.74E-02	PLCD3,RRAS2,RRAS,PARP12,PARP9,PARP14
Remodeling of Epithelial Adherens	1.39E+00	7.58E-02	MET,CTNNA2,TUBB6,TUBB4A,MAPRE3

Junctions			
G Beta Gamma Signaling	1.39E+00	6.00E-02	RRAS2,GRB2,RRAS,GNA12,CAV1,GNG10
Apoptosis Signaling	1.39E+00	6.52E-02	RRAS2,CAPNS1,RRAS,CASP2,CAPN2,BCL2L11
D-myo-inositol (1,4,5)-Trisphosphate Biosynthesis	1.38E+00	1.11E-01	PLCD3,PIP4K2B,PIP4K2A
Superpathway of Cholesterol Biosynthesis	1.38E+00	1.11E-01	HADHB,HMGCS1,HADHA
ERK/MAPK Signaling	1.38E+00	5.03E-02	ITGB1,ELF4,H3F3A/H3F3B,RRAS2,ARAF,GRB2,RRAS,PPP1CB,TLN1,RAP1A
Hereditary Breast Cancer Signaling	1.37E+00	5.88E-02	RAD51,RRAS2,RRAS,HDAC7,CDK6,CDK4,RFC1
Virus Entry via Endocytic Pathways	1.37E+00	6.52E-02	ITGB1,RRAS2,RRAS,CAV1,ITGB8,ITGB7
Thrombin Signaling	1.35E+00	5.08E-02	PLCD3,ADCY9,RHOG,RRAS2,GRB2,RRAS,GNA12,ARHGEF16,PPP1CB,GNG10
Salvage Pathways of Pyrimidine Ribonucleotides	1.33E+00	6.59E-02	MAP2K6,NME4,ARAF,CDK6,CDK4,MAP2K3
Synaptic Long Term Potentiation	1.32E+00	6.03E-02	PLCD3,RRAS2,PPP3CB,RRAS,GRM3,PPP1CB,RAP1A
PPARα/RXRα Activation	1.31E+00	5.17E-02	MAP2K6,PLCD3,ADCY9,RRAS2,GRB2,RRAS,TGFB1,MAP2K3,ACVR1B

5B: Gene ontology analysis of miR-124-3p effect on gene expression

Description	Term_ID	log10 p-value	
regulation of cellular component movement	GO:0051270	-1.5229	
nucleoside metabolic process	GO:0009116	-1.3028	
response to endoplasmic reticulum stress	GO:0034976	-1.3872	
cell junction organization	GO:0034330	-1.3028	
regulation of phosphate metabolic process	GO:0019220	-1.5229	
vesicle-mediated transport	GO:0016192	-1.4724	
regulation of transferase activity	GO:0051338	-1.3872	
glycosyl compound metabolic process	GO:1901657	-1.3516	
establishment of protein localization	GO:0045184	-1.3872	
regulation of phosphorus metabolic process	GO:0051174	-1.5229	
protein phosphorylation	GO:0006468	-1.3883	
positive regulation of phosphorus metabolic process	GO:0010562	-1.3872	
positive regulation of phosphate metabolic process	GO:0045937	-1.3872	
regulation of protein phosphorylation	GO:0001932	-1.3883	
regulation of kinase activity	GO:0043549	-1.3872	
regulation of protein kinase activity	GO:0045859	-1.3028	
regulation of phosphorylation	GO:0042325	-1.4724	
regulation of protein modification process	GO:0031399	-1.4498	
regulation of cellular protein metabolic process	GO:0032268	-1.3883	

Supplementary Table 6. Validation set patient cohorts

Specimen Type	Sex	Age at diagnosis	Laterality	Diagnosis	Max tumor size (cm)	T	N	M	Fuhrman Grade	Applied tissue
Kidney	M	61	R	ccRCC	9	T3a	NX	MX	2	FFPE
Kidney	F	41	L	ccRCC	7.5	T2a	NX	M1	2	FFPE
Kidney	M	52	L	ccRCC	6.5	T3b	N1	NX	2	FFPE
Kidney	M	57	NA	ccRCC	5.5	NA	NA	NA	4	FFPE
Kidney	NA	NA	NA	ccRCC	NA	NA	NA	NA	NA	FFPE
Kidney	M	NA	NA	ccRCC	5.8	NA	NA	NA	2	FFPE
Kidney	M	NA	NA	ccRCC	5.5	NA	NA	NA	2	FFPE
Kidney	M	NA	NA	ccRCC	6.5	NA	NA	NA	3	FFPE
Kidney	F	42	L	ccRCC	8.5	T2a	N1	MX	4	FFPE
Kidney	F	69	L	ccRCC	9	T4	N0	M1	2	FFPE
Kidney	M	70	R	ccRCC	13.5	T3a	n1	MX	2	FFPE
Kidney	M	48	L	ccRCC	8	T2a	NX	M1	3	FFPE
Kidney	M	68	R	ccRCC	NA	T3a	NX	Mx	3	FFPE
Kidney	M	51	R	ccRCC	6	T3b	NX	Mx	3	FFPE
∟ matched metastasis: Peritoneal Node				metastatic ccRCC						FFPE
Kidney	M	74	R	ccRCC	7.5	T3a	N1	Mx	4	FFPE
∟ matched metastasis: Hilar Node				metastatic ccRCC						FFPE
Kidney	M	72	R	ccRCC	1.5	T1a	NA	M1	NA	FFPE
∟ matched metastasis: Adrenal Gland				metastatic ccRCC						FFPE
Kidney	M	44	R	ccRCC	8	T3a	NX	NA	2	FFPE
∟ matched metastasis: Skin				metastatic ccRCC						FFPE
Kidney	M	74	R	ccRCC	6	T3a	Nx	M1	3	FFPE
∟ matched metastasis: Skin				metastatic ccRCC						FFPE
Kidney	M	59	L	ccRCC	18	T3a	N0	M1	4	FFPE
Kidney	M	56	L	ccRCC	9.5	T2a	Nx	M1	3	FFPE
∟ matched metastasis				metastatic ccRCC						FFPE
Kidney	F	62	R	ccRCC	8	T2a	Nx	M1	3	FFPE
Kidney	M	55	L	ccRCC	7	T1b	NA	NA	NA	FFPE
Kidney	F	72	L	ccRCC	4	T1a	NA	NA	2	FFPE
Kidney	M	67	L	ccRCC	2.5	T1a	NA	NA	1	FFPE
Kidney	F	58	R	ccRCC	4	T1a	N0	NA	2	FFPE
Kidney	F	60	L	ccRCC	5	T1b	NA	NA	2	FFPE
Kidney	F	25	L	ccRCC	6.5	T1b	NA	NA	1	FFPE
Kidney	M	62	L	ccRCC	7.5	T2	NA	NA	3	FFPE
Kidney	F	69	R	ccRCC	5.2	T1b	NA	NA	2	FFPE
Kidney	M	42	L	ccRCC	9	T2	NA	NA	2	FFPE
Kidney	M	61	R	ccRCC	5.5	T3	NA	NA	4	FFPE
Kidney	M	70	L	ccRCC	3.5	T1a	NA	NA	2	FFPE
Kidney	F	46	R	ccRCC	9.7	T2b	NA	NA	4	FFPE
Kidney	M	39	L	ccRCC	5	T1b	NA	NA	3	FFPE
Kidney	M	87	L	ccRCC	7.5	T3	NA	NA	2	FFPE
Kidney	F	80	L	ccRCC	7	T1b	N0	NA	1	FFPE

Kidney	F	82	L	ccRCC	7	T1b	NA	M1	3	FFPE
Kidney	M	60	L	ccRCC	6	T1b	NA	NA	3	FFPE
Kidney	F	36	R	ccRCC	2.4	T1a	NA	NA	2	FFPE
Kidney	M	74	R	ccRCC	1.5	T1a	NA	NA	3	FFPE
Kidney	F	69	L	ccRCC	3.5	T1a	NA	NA	2	FFPE
Kidney	M	75	R	ccRCC	4.5	T3	NA	NA	1	FFPE
Kidney	F	73	L	ccRCC	3	T1a	NA	NA	2	FFPE
Kidney	F	69	R	ccRCC	2.8	T1a	NA	NA	2	FFPE
Kidney	M	67	L	ccRCC	10	T3a	NA	NA	3	FFPE
Kidney	M	77	L	ccRCC	2.5	T1a	NA	NA	2	FFPE
Kidney	M	64	R	ccRCC	7.5	T3b	NA	NA	3	FFPE
Kidney	F	68	L	ccRCC	4	T1a	NA	NA	2	FFPE
Kidney	F	62	R	ccRCC	3	T1a	NA	NA	3	FFPE
Kidney	M	73	L	ccRCC	3	T1a	NA	NA	3	FFPE
Kidney	M	43	R	ccRCC	3.2	T1a	NA	NA	3	FFPE
Kidney	M	74	R	ccRCC	3	T1a	NA	NA	2	FFPE
Kidney	M	64	R	ccRCC	3.5	T1a	NA	NA	2	FFPE
Kidney	M	55	L	ccRCC	20	T4	NA	NA	4	FFPE
Kidney	M	63	L	ccRCC	13	T3a	NA	NA	4	FFPE
Kidney	M	64	R	ccRCC	11.5	T2b	NA	NA	4	FFPE
Kidney	M	55	L	ccRCC	10	T2b	NA	NA	4	FFPE
Kidney	M	64	L	ccRCC	8	T3a	N1	MX	4	FFPE
Kidney	M	51	R	ccRCC	7.3	T2a	Nx	MX	4	FFPE
Kidney	F	53	R	ccRCC	6.3	T3a	N1	MX	3	FFPE
Kidney	M	65	L	ccRCC	10.5	T2b	NX	MX	2	FFPE
Kidney	M	52	R	ccRCC	10.5	T3a	NX	MX	4	FFPE
Kidney	M	48	R	ccRCC	1.4	T1a	NA	M0	1- 2	fresh frozen
Kidney	M	68	R	ccRCC	8	T2	NA	M0	1	fresh frozen
Kidney	M	64	L	ccRCC	8	T2	NA	M0	2	fresh frozen
Kidney	M	72	R	ccRCC	4	T3b	NA	NA	1-2/4	fresh frozen
Kidney	M	70	L	ccRCC	3	T1a	NA	M0	2	fresh frozen
Kidney	M	32	R	ccRCC	2	T1a	NA	NA	2/4, 1/4	fresh frozen
Kidney	M	37	R	ccRCC	2.5	T1a	NA	M0	2	fresh frozen
Kidney	M	69	R	ccRCC	2.5	T1a	NA	M0	2	fresh frozen
Kidney	M	80	NA	ccRCC	7	T1b	NA	MX	3	fresh frozen
Kidney	M	29	L	ccRCC	3.5	T1	NA	M0	2	fresh frozen
Kidney	M	45	R	ccRCC	2.7	T1a	NA	MX	3	fresh frozen
Kidney	M	65	L	ccRCC	3.3	T1	NA	M0	1	fresh frozen
Kidney	M	59	R	ccRCC	7.4	T3a	NA	MX	3	fresh frozen
Kidney	M	58	R	ccRCC	7	T1b	NA	MX	2	fresh frozen
Kidney	M	74	L	ccRCC	4	T1a	NA	NA	2	fresh frozen
Kidney	M	53	L	ccRCC	8.6	T2	NA	MX	3	fresh frozen
Kidney	M	70	R	ccRCC	3	T1	NA	M0	1	fresh frozen
Kidney	M	72	L	ccRCC	4.7	T1b	NA	MX	3	fresh frozen
Kidney	M	62	L	ccRCC	5.5	T3a	NA	NA	3-4	fresh frozen
Kidney	M	67	R	ccRCC	4	T1a	NX	MX	3	fresh frozen

Kidney	M	65	R	ccRCC	4	T1	NA	NA	2	fresh frozen
Kidney	M	67	R	ccRCC	4.5	T1b	NX	MX	3	fresh frozen
Kidney	M	58	R	ccRCC	6	T3a	NX	MX	3	fresh frozen
